# Genome-Wide Analyses Reveal Footprints of Divergent Selection and Drought Adaptive Traits in Synthetic-Derived Wheats

**DOI:** 10.1534/g3.119.400010

**Published:** 2019-04-24

**Authors:** Fakiha Afzal, Huihui Li, Alvina Gul, Abid Subhani, Ahmad Ali, Abdul Mujeeb-Kazi, Francis Ogbonnaya, Richard Trethowan, Xianchun Xia, Zhonghu He, Awais Rasheed

**Affiliations:** * Atta-ur-Rahman School of Applied Biosciences, National University of Sciences and Technology, H-12 Islamabad, Pakistan; † Institute of Crop Sciences, Chinese Academy of Agricultural Sciences (CAAS) and; ‡ International Maize and Wheat Improvement Centre (CIMMYT), c/o CAAS, Beijing 100081, China; § School of Integrative Plant Science, Cornell University, Ithaca, NY 14853; ** Barani Agricultural Research Institute (BARI), Chakwal, Pakistan; †† Center for Plant Science and Biodiversity, University of Swat, Swat, Paksitan; ‡‡ Texas A&M University, Amarillo, TX 79106; §§ Grains Research & Development Corporation, Kingston, Australia; *** Plant Breeding Institute, School of Life and Environmental Sciences, The University of Sydney, Sydney 2006, Australia, and; ††† Department of Plant Sciences, Quaid-i-Azam University, Islamabad 45320, Pakistan

**Keywords:** synthetic-derived wheats (SYN-DER), genome-wide association studies (GWAS), single nucleotide polymorphisms (SNPs), haplotype analysis, selective sweeps

## Abstract

Crop-wild introgressions have long been exploited without knowing the favorable recombination points. Synthetic hexaploid wheats are one of the most exploited genetic resources for bread wheat improvement. However, despite some QTL with major effects, much less is known about genome-wide patterns of introgressions and their effects on phenotypes. We used two genome-wide association approaches: SNP-GWAS and haplotype-GWAS to identify SNPs and haplotypes associated with productivity under water-limited conditions in a synthetic-derived wheat (SYN-DER) population. Haplotype-GWAS further enriched and identified 20 more genomic regions associated with drought adaptability that did not overlap with SNP-GWAS. Since GWAS is biased to the phenotypes in the study and may fail to detect important genetic diversity during breeding, we used five complementary analytical approaches (*t*-test, Tajima’s D, nucleotide diversity (π), *Fst*, and EigenGWAS) to identify divergent selections in SYN-DER compared to modern bread wheat. These approaches consistently pinpointed 89 ‘selective sweeps’, out of which 30 selection loci were identified on D-genome. These key selections co-localized with important functional genes of adaptive traits such as *TaElf3-D1* (1D) for earliness *per se* (Eps), *TaCKX-D1* (3D), *TaGS1a* (6D) and *TaGS-D1* (7D) for grain size, weight and morphology, *TaCwi-D1* (5D) influencing drought tolerance, and *Vrn-D3* (7D) for vernalization. Furthermore, 55 SNPs and 23 haplotypes of agronomic and physiological importance such as grain yield, relative water content and thousand grain weight in SYN-DER, were among the top 5% of divergent selections contributed by synthetic hexaploid wheats. These divergent selections associated with improved agronomic performance carry new alleles that have been introduced to wheat. Our results demonstrated that GWAS and selection sweep analyses are powerful approaches for investigating favorable introgressions under strong selection pressure and the use of crop-wild hybridization to assist the improvement of wheat yield and productivity under moisture limiting environments.

Genetic variation in food crops has been successfully exploited through conventional breeding over the last century, resulting in 0.8–1.2% annual genetic yield gain in five major food crops including wheat (Ray *et al.*, 2013). However, this rate of genetic gain is insufficient to achieve the 100% increase in production target needed to meet global demand for food, feed, and fiber by 2050 (Tilman *et al.*, 2011). A steady increase in genetic gain is required to fulfill the demands of the 2% yearly increase in world population. Such an increase is challenging because there is little scope to improve harvest index due to the genetic bottleneck introduced by modern crop improvement practices reliant on few parents. Furthermore, weather extremes and continuously shrinking arable land are additional challenges to increasing yield and yield stability in diverse environments (Xu *et al.*, 2017). In conventional wheat breeding, a limited proportion of the genetic variation available in the bread wheat genepool for abiotic and biotic stress tolerance and quality attributes has been explored to maximize genetic gain. Some estimate that 69% of genetic diversity has been lost through domestication and modern breeding (Reif *et al.*, 2005). Re-introducing lost genetic diversity from wheat wild relatives by integration of molecular genetics and conventional breeding technologies is one way to bridge this gap (Rasheed *et al.*, 2018).

Exploiting crop wild relatives for quantitative traits is challenging because the germplasm may be too diverse to be used directly and a pre-breeding process will be required. Therefore, genetic approaches such as large-scale and systematic identification and characterization of quantitative trait loci (QTL) through GWAS could help to identify alleles or haplotypes that may not exist in crop cultivars but could be incorporated into the existing elite gene pool (Bevan *et al.*, 2017). Synthetic hexaploid wheat (SHW) developed by hybridizing *Aegilops tauschii*, D-genome donor to bread wheat (*Triticum aestivum* L.), is a source of genetic diversity and favorable alleles for tolerance to biotic and abiotic stresses (Börner *et al.*, 2015; Mujeeb-Kazi *et al.*, 1996; Ogbonnaya *et al.*, 2013). Synthetic hexaploid wheat and their advanced derivatives (SYN-DERs) have potential to improve drought- and heat-adaptive mechanisms in bread wheat (Afzal *et al.*, 2017; Reynolds *et al.*, 2005). SYN-DERs have outperformed their recurrent parents under drought stress and have proved to be high yielding compared to non-synthetic wheat in various studies (Jafarzadeh *et al.*, 2016; Lopes and Reynolds 2011; Tang *et al.*, 2017). Despite several GWAS and QTL mapping studies available in synthetic hexaploid wheats (Ogbonnaya *et al.*, 2013; Rasheed *et al.*, 2018), underpinning the recombinations favoring a yield-advantage in SYN-DER is lacking. Unlocking the genetic potential of genetic resources requires whole-genome sequence coverage through next-generation sequencing; however large wheat genome-size (∼16Gb) and incomplete reference genome makes this unfeasible. Alternatively, single nucleotide polymorphism (SNPs) arrays can be used to define genomic regions associated with quantitative traits due to their dense genome coverage (Akhunov *et al.*, 2009; Rasheed *et al.*, 2017; Wang *et al.*, 2014).

SNPs have been extensively used in mapping experiments to identify the genetic loci associated with important agronomic phenotypes. In human genetic studies, haplotype blocks combining two or more SNPs in strong LD are more informative than single bi-allelic SNPs (Stephens *et al.*, 2001). Lorenz *et al.* (2010) defined haplotypes by several analytical procedures in barley and confirmed that the distribution of QTL alleles in nature was unlike the distribution of marker variants, and hence utilizing haplotype information could capture associations that would elude single SNPs. Haplotype based GWAS analyses are still rare in wheat except for few studies (Hao *et al.*, 2017; Jordan *et al.*, 2015), and have shown promise in Brassica (Wu *et al.*, 2016), barley (Lorenz *et al.*, 2010) and other crop species (Qian *et al.*, 2017). Bevan *et al.* (2017) argued that haplotypes in progenitors and wild relatives of crops contain a broad range of genetic variation and identification of haplotypes with fewer deleterious alleles and improved phenotypes could accelerate genetic gain in crop improvement. Such GWAS approaches either with bi-allelic SNPs or haplotypes could be combined with genome-wide selective sweeps which identifies selection signatures that are beneficial for crop adaptation. Crop breeding selects favorable alleles and retains them in new cultivars. These signatures of selection can be detected by a cross-population comparison approach (Chen *et al.* 2010). Several lines of evidence have shown that that genomic regions that exhibit selection signatures are also enriched for genes associated with biologically important traits (Xie *et al.* 2015). Therefore, detection of selection signatures is emerging as an additional approach to identify and validate novel gene-trait associations (Cadzow *et al.* 2014).

This study aimed to identify the chromosomal regions under divergent selection in SYN-DER wheat by comparison to modern bread wheat cultivars widely grown in the same environments. We used two GWAS strategies (SNP-GWAS and haplotype-GWAS) to identify marker-trait associations under divergent selection in SYN-DERs.

## Materials and Methods

### Germplasm and phenotyping

A total of 240 hexaploid wheat accessions including 171 SYN-DER wheats and 69 modern bread wheat cultivars and advanced lines were assessed (Table S1). Among the modern bread wheat cultivars, 32 were recurrent bread wheat parents used in the development of SYN-DER. These SYN-DERs were developed by crossing primary SHWs with advanced lines and elite cultivars of Pakistan and CIMMYT (see pedigrees in Table S1). More than 800 SYN-DERs were developed initially at the National Agriculture Research Center (NARC), Pakistan. This was reduced to 171 following re-selection for better agronomic characteristics. Field trials were conducted at two locations during the 2014-15 and 2015-16 cropping seasons at the Barani Agriculture Research Institute (BARI), Chakwal (33°40^/^38^//^N 72°51^/^21^//^E, 498m asl), Pakistan and the National Agriculture Research Center (NARC), Islamabad (33°43^/^N 73°04^/^E, 579 asl), Pakistan. At each location/year, field trials were conducted in two water regimes *i.e.*, well-watered (WW) and water-limited (WL) conditions. Chakwal is a rain-fed, semi-arid area lies at the beginning of the Potohar plateau. The deep, well-drained soil comprises moderately fine textured particles. It is slightly calcareous, non-saline, with pH 7.6 and an electrical conductivity of 0.32 dS/m (Islam *et al.* 2012). Average rainfall during cropping season in 2014 was 20 mm and was 22.14 mm in 2015. A field experiment with an alpha lattice design was set up in both WW and WL conditions. The first environmental condition was established as well-watered (WW) in which all genotypes with two replications were planted in the field. Three irrigations were given to well-watered (WW) plants, and soil moisture was maintained at field capacity (100%) until harvest. The second environmental condition was water-limited (WL) in which all genotypes were planted with two replications in a polyethylene tunnel supported by an iron frame to provide shelter from precipitation. A 1-m deep ditch surrounded the tunnel to prevent any seepage of rainwater. Irrigation was stopped at the end of tillering through the completion of the flowering stage. The crop growth stages were determined using the Zadoks scale (Zadoks *et al.* 1974). Two 2-m long rows were sown with each genotype with two replications, maintaining an inter-row spacing of 30 cm for both treatments. The sowing date was November 20 in both years. To establish uniform stands, we sowed 30 viable seeds of variable kernel mass with the help of small plot grain drill for each row. To ensure sufficient nutrition, standard agronomic practices were followed.

Phenotypic traits recorded at each location included chlorophyll content index (CCI), total chlorophyll (Chl), canopy temperature (CT), number of grains per spike (GS), grain yield (GY), plant height (PH), proline, relative water contents (RWC), shoot dry weight (SDW), shoot fresh weight (SFW), spike length (SL), superoxide dismutase (SOD), sugar contents (Sugar), thousand grain weight (TGW), and tillers per plant (TP). Specific suffixes were provided to each trait according to the water treatment *e.g.*, GY_WW_ (GY in well-watered treatment), GY_WL_ (GY in water-limited treatment), and GY_DIFF_ (GY for difference in well-watered and water-limited treatments).

A chlorophyll meter of model CCM200 plus was used to measure chlorophyll content index. Similarly, canopy temperature was measured using an infrared thermometer IRT206 at booting stage (Z45) (Zadoks *et al.* 1974). Plant height was measured from the base of the plant to the spike excluding awns at Z96 stage. Similarly, number of tillers per plant (TP) and spike length (SL) excluding awns were recorded for ten spikes from each replicate. Number of grains per spike were calculated after harvesting on the same 10 random spikes and thousand grain weight was recorded.

For biochemical analysis, fresh leaf samples were collected at the onset of flowering stage (Z60) (Zadoks *et al.* 1974). Sugar contents or soluble sugar in leaves were estimated (Dobois *et al.* 1956). For this purpose, 0.5 g of fresh leaf tissues were taken with 10 mL of ethanol and were heated at 80° in for one hour with continuous shaking. One mL phenol (18%) was added to the 0.5 ml of pre-heated extract and incubated for one hour. After this, 2.5 mL of concentrated H_2_S0_4_ was added and vortexed. The extract was used to measure absorbance at 420 nm by a UV spectrophotometer (Biochem-2100) and the concentration of soluble sugar was measured using a standard glucose curve.

Total chlorophyll was measured using dimethyl sulphoxide (DMSO) (Hiscox and Israelstam 1979). Briefly, 0.5 gram of leaf tissues were taken in a preheated tube filled with 6 mL DMSO. Material was heated in a hot water bath at 65° for 35-40 min. Then extract was transferred to a new tube and DMSO was added until total volume reaches 10 mL. Finally, total chlorophyll contents were measured by recording absorbance of the extract at 660 nm using RS-1100 equipment and fitting into the equation of Arnon (1949).

Proline was measured following protocol of Bates *et al.* (1973). For this, 0.3 g of fresh leaf sample was homogenized with 5 mL of sulfo-salyclic acid (3%) in a test tube. Then two mL of mixture was added to 2 mL of glacial acetic acid and 2 mL of ninhydrin and incubated for one hour at 100°. The reaction was stopped with ice and then four mL toluene was added to reaction mixture. The mixture absorbance value recorded at 420 nm using UV spectrophotometer. A standard curve was made using authentic proline and proline was calculated as μmol/g.

The concentration of superoxide dismutase (SOD) in leaf was measured with following protocol. First 0.5 g of fresh wheat leaf samples were ground in a chilled mortar and pestle with 10 mL of chilled phosphate buffer (50 mM) in an ice bath. The mixture was centrifuged at 13,000 RPM at 4° for 25 min and was filtered in four layers of cheesecloth. The supernatant obtained was used to assay SOD activity (Beauchamp and Fridovich 1971; Giannopolistis and Ries 1977). The extract obtained was illuminated under 40 W fluorescent lamps at 25° with 100 µL of 1.3 µM riboflavin and 3 mL of 0.1 M SOD buffer for 8 min until a dark color appeared. For the samples from well-water conditions, the same protocol was followed but in darkness. A blank check without any leaf sample was prepared. The absorbance of all three mixtures was measured at 560 nm in the UV spectrophotometer. The concentration of SOD is the quantity of enzyme preventing 50% of photoreduction by nitroblue tetrazolium (NBT) in contrast to the sample mixture that lacks plant material.

Relative water content (RWC) was measured as per method devised by Bar and Weatherly (1962) with slight modifications. Three fully expanded flag leaves from randomly chosen plants in each plot were collected from both environmental conditions (WW and WL) at Z45 of booting stage (Zadoks *et al.* 1974). Top and bottom of the leaves with any dead tissue was removed off to leave a 5 cm mid-section. All samples were then immediately placed in pre-weighed air tight falcon tubes to stop any moisture loss/gain from the system. Tubes were then transferred to a cooled and insulated container (at around 10°–15°; but not frozen). All tubes with sample were then weighed and recorded under TW + FW. Tubes were then filled with 1 mL of distilled water and placed in refrigerator (4°) for 24h. This will make leave turgid. After carefully blotting with dry paper to remove moisture, hydrated leaves were weighed and recorded under TW. All leaf samples were then transferred to labeled envelopes to oven (70°) for 24h. Samples were then reweighed and recorded under DW. RWC was measured by using formulae;

Leaf RWC(%)=[(FW-DW)/(TW-DW)]×100

where FW = leaf fresh weight, TW = leaf turgid weight, and DW = leaf dry weight.

### Genotyping and analysis of genotyping datasets

Five viable seeds of each genotype were planted in 5 cm diameter pots. DNA was extracted per the CIMMYT Molecular Genetics Manual (Dreisigacker *et al.*, 2013) from fresh leaf samples of 25-day-old seedlings. DNA samples (50–100 ng/µl per sample) were sent to Department of Primary Industries, Victoria, Australia for genotyping with high-density wheat 90K infinium SNP array (Wang *et al.*, 2014). Genotypic clusters for every SNP were determined following the manual for Genome Studio version 1.9.4 with the polyploid clustering version 1.0.0 (Illumina; http://www.illumina.com), based on the data from all the genotypes.

The physical position of SNPs on the wheat genome were determined based on IWGSC RefSeq version 1.0 (IWGSC 2018). Polymorphism information content (PIC) was used to determine genetic diversity at each locus. Markers with ambiguous SNP calls, that were monomorphic or with missing values of more than 20% and less than 5% MAF (minor allele frequency) were removed from the dataset. Genetic similarities between wheat genotypes were estimated using PowerMarker v.3.0 with a Dice coefficient based on the proportion of shared alleles (Liu and Muse 2005).

Population structure was assessed with 1000 unlinked SNP markers using STRUCTURE software 2.3.3, which implements a model-based Bayesian cluster analysis. Structure matrix (Q-matrix) was formed by organization of all population entries into clusters. An assumed number of subpopulation (*k*) ranging from 1 to 10 was evaluated using 100,000 burn-in iterations followed by 500,000 recorded Markov-Chain iterations. Robustness (sampling variance) of inferred population structure was estimated by carrying out 10 independent runs for each *k*. The optimum number of subpopulations was determined utilizing ADHOC statistics Δ*k* based on the rate of change in log probability of data between successive *k* (Evanno *et al.* 2005).

Genome-wide linkage disequilibrium (LD) was evaluated across A, B and D genomes. Using TASSEL v.5.0, the LD parameter r^2^ was calculated for all the pairwise markers which could be aligned to the consensus map for both entire panel and model-based subgroups. To examine LD due to the physical linkage in particular, the critical r^2^ value (Breseghello and Sorrells 2006) was investigated. This was calculated by taking the 95^th^ percentile of the square root transformed r^2^ data of unlinked markers (Breseghello and Sorrells 2006). An r^2^ beyond the critical r^2^ value was declared to be caused by genetic linkage. Only the LD pairs significant at (*P* < 0.001) were included in LD decay plots and stacked bar plots.

### Statistical analysis of phenotyping datasets

The phenotypic data were collected from the two locations over two consecutive years under well-watered and water-limited conditions. The data were averaged for both years x locations leading to two datasets *i.e.*, well-watered and water-limited for each trait. ANOVA was used to test the statistical significance of different sources of variation for each of the nine traits. In the ANOVA model, phenotypic effect was partitioned into overall mean, treatment effect, replication (*i.e.*, block) within environment (year and location combination) effect, genotypic effect, environment effect, genotype by environment effect, genotype by treatment effect, and random error effect. Let *y*_lijk_ be the observed value of a trait of interest for the *i*^th^ accession in the *k*^th^ replication under the *j*^th^ environment (equivalent to location and year in this study) and the *l*^th^ treatment. The linear model used in ANOVA is therefore,

$$y_{\mathrm{lijk}}=\mu +D_{l}+R_{k/j}+G_{i}+E_{j}+GE_{\mathrm{ij}}+GD_{il}+\varepsilon_{\mathrm{lijk}}\text{,}$$ [1]

where *l* = 1, 2, ..., *L* (*L* = 2 for well-watered and water-limited treatments), *i* = 1, 2, ..., *n* (*n* = 203), *j* = 1, 2, ..., *e* (*e* = 4 with two locations and two years), *k* = 1, 2, ..., *r* (*r* = 2), is overall mean of the whole population, *R*_k/j_ is the *k*^th^ replication effect in the *j*^th^ environment, *G*_i_ is genotypic effect of the *i*^th^ accession, *E*_j_ is environmental effect of the *j*^th^ environment, *GE*_ij_ is interaction effect between the *i*^th^ accession and the *j*^th^ environment, *GD*_il_ is interaction effect between the *i*^th^ accession and the *l*^th^ treatment, and *ε*_lijk_ is random error effect which was assumed to be normally distributed with a mean of zero, and variance $\sigma_{\varepsilon }^{2}$. The ANOVA described above was implemented with the GLM procedure in SAS software (SAS Institute, Cary, NC, 2007).

The BLUP of genotypic value for each accession under each water treatment was used as the phenotype for all subsequent comparisons. BLUPs were calculated as follows: the observed value of trait was defined as *y*_ijk_ for the *i*^th^ accession in the kth replication in the *j*^th^ environment (equivalent to location and year in this study). The mixed model used for BLUP was therefore,

$$y_{\mathrm{ijk}}=\mu +R_{k/j}+G_{i}+E_{j}+GE_{\mathrm{ij}}+\varepsilon_{\mathrm{ijk}}\text{, and }\varepsilon_{ijk}∼N(0,\;\sigma_{\varepsilon }^{2})\text{,}$$ [2]

where *i* = 1, 2, ..., *n* (*n* = 203), *j* = 1, 2, ..., *e* (*e* = 4 with two locations and two years), *k* = 1, 2, ..., *r* (*r* = 2), μ, *R_k/j_*, *G_i_*, *E_j_*, and *GE_ij_* were the same as the description above. Except for, all the effects were viewed as random effects following the normal distributions $R_{k/j}∼N(0,\;\sigma_{R}^{2})$, $G_{i}∼N(0,\;\sigma_{G}^{2})$, $E_{j}∼N(0,\;\sigma_{E}^{2})$, and $GE_{ij}∼N(0,\;\sigma_{GE}^{2})$, where $\sigma_{R}^{2}$, $\sigma_{G}^{2}$, $\sigma_{E}^{2}$, and $\sigma_{GE}^{2}$were the variances explained by replication, genotype, environment, and genotype by environment interaction, respectively. The BLUPs were calculated with the MIXED procedure in SAS software (SAS Institute, Cary, NC, 2007).

### Genome-wide association studies and selective sweeps

#### Genome-wide association analysis using SNP markers:

For marker-trait associations (MTAs), a mixed linear regression (MLM) model controlling both population structure (Q matrix) and kinship matrix (K matrix) was applied in TASSEL Standalone v.5.0 (Yu and Buckler 2006). Quantile-quantile plots of estimated *vs.* observed P values from MTAs were also produced and deviations from the expectation demonstrated that statistical analysis might cause spurious associations. Bonferroni corrections were applied, and a P-value of 10^−5^ was defined as the threshold for significant MTAs. SNPs with P-values in the range of 10^−3^-10^−4^ for one trait and P-values <10^−5^ for another trait were also reported. The LD decay distance at r^2^ = 0.1 was used as the support interval to avoid multiple significance within one LD block.

#### Genome-wide haplotype blocks and haplotype-GWAS:

Genome-wide haplotype blocks were constructed using PLINK with the default parameters as used by Haploview 4.2 software package (http://www.broadinstitute.org/haploview/haploview). The package defined haplotype blocks based on 4 gametes, and provided the number of haplo-groups and their genetic length (bp) for each block, as well as the number of tag SNPs based on solid spine of linkage disequilibrium (LD) (Extend spine if D′>0.8). In brief, this meant that the first and the last marker in a block were in strong LD with the intermediate markers that were not necessarily in LD with each other. All the haplotype blocks consisting of two haplo-groups were removed to rule out perfect LD and similarity to SNP polymorphism. Haplotype frequency was calculated using a custom Perl script. If a haplotype block is only observed in one group (SYN-DER or BW) but not in the other group, it is considered to be a group-specific block. If a haplotype block was simultaneously detected in both groups, the haplotype block was considered to be a common block. Haplotype-GWAS was performed using the linear regression procedure implemented in PLINK (Purcell *et al.*, 2007), where phenotype BLUPs were regressed on the number of haplo-groups of a particular haplotype using PLINK linear option, including population structure as covariate. The P-value threshold of 10^−5^ was defined to declare haplo-groups associated with phenotypes.

#### Identification of loci Under selection for selective sweeps:

Five statistical methods/parameters were used to detect the loci under selection. (1) Difference in allele frequencies for the *m*^th^ marker locus between the bread wheat (BW) set and the SYN-DER set were tested by Student’s *t*-test:

$$t=\frac{f_{1}-f_{2}}{\sqrt{\left(\frac{1}{2n_{1}}+\frac{1}{2n_{2}})f_{\exp}(1-f_{\exp}\right)}}$$

where $f_{\exp}=\frac{f_{1}n_{1}+f_{2}n_{2}}{n_{1}+n_{2}}$, *f*_1_ and *f*_2_ were the allele frequencies of a specific marker locus in the BW and SYN-DER sets, respectively, and *n*_1_ and *n*_2_ were the sample sizes in the BW and SYN-DER sets, respectively. The population-specific alleles were determined for each subpopulation based on zero allele frequency in one subpopulation and non-zero in another subpopulation; and different allele frequencies between two subpopulations at significance level *P* < 0.001.

(2) *F*st was calculated for individual SNPs by

$$F_{st}=\frac{\sigma_{f}^{2}}{\bar{f}(1-\bar{f})}=\frac{2\left[\sum_{l=1}^{2}w_{l}{\left(f_{l}-\bar{f}\right)}^{2}\right]}{\bar{f}(1-\bar{f})}$$

where $\bar{f}$ and $\sigma_{f}^{2}$ were the mean and variance of allele frequencies, respectively, $w_{1}=\frac{n_{1}}{n_{1}+n_{2}}$, and $w_{2}=\frac{n_{2}}{n_{1}+n_{2}}$ (Weir and Cockerham 1984). VCFtools (https://vcftools.github.io/index.html) were used to calculate *F*st with a sliding window of 100 kb and a step size of 10 kb (Schmutz *et al.*, 2014) over the whole genome, and the regions with the top 5% of *F*st values were regarded as highly diverged across the two groups.

(3) A search for loci under selection using genome-wide association of eigenvectors was implemented by EigenGWAS (Chen *et al.*, 2016) requiring three steps. First, a genetic relationship matrix was generated for the 235 accessions, 69 from BW set and 171 from SYN-DER panel. Assuming that $X_{i}={\left(x_{i1},x_{i2},...,x_{iM}\right)}^{T}$ was a vector of genotype for the *i*^th^ individual, where *x* was the number of the alleles and *M* was the number of marker loci, the genetic relationship matrix ***A*** for each pair of 240 accessions was calculated by

$$A_{ij}=\frac{1}{M}\sum_{m=1}^{M}\frac{\left(x_{im}-2f_{m})(x_{jm}-2f_{m}\right)}{2f_{m}(1-f_{m})}$$

where *f*_m_ is the allele frequency for the *m*^th^ marker locus. Second, the principle component analysis (PCA) was conducted on the ***A*** matrix (Price *et al.* 2006). Then matrix **E** with dimension N×C was retained, where E_c_ was the eigenvector corresponding to the *c*^th^ largest eigenvector. In this study, *c* was set as 10 to calculate the top 10 eigenvalues and eigenvectors. Finally, single marker regression on E_c_ was conducted to estimate the effect of each marker.

Further, (4) Tajima’s D, and (5) nucleotide diversity (π) relative to the founder population (π_SYN-DER_/π_BW_) were calculated using VARISCAN version 2.0 with a sliding window of 100 kb and a step size of 10 kb.

### Data availability

The authors affirm that all data necessary for confirming the conclusion of this article are represented fully within the article and its tables and figures. Supplemental material available at FigShare: https://doi.org/10.25387/g3.7356923.

### Ethics statement

The field trials were permitted by National Agriculture Research Center (NARC), Islamabad, Pakistan and Barani Agriculture Research Institute (BARI), Chakwal, Pakistan. The remaining experimentation does not require any ethical statement.

## Results

**Figure 1:**
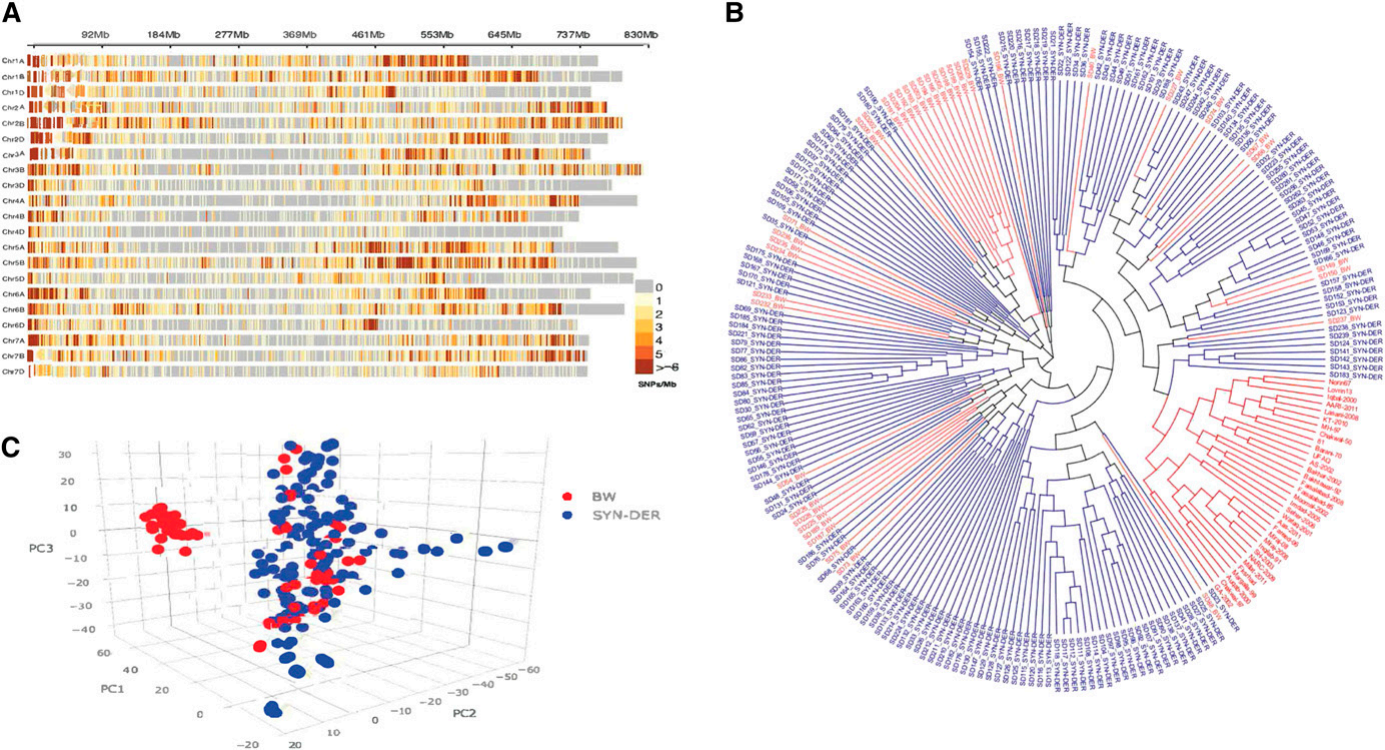
a) Genome-wide SNP marker density on each bread wheat chromosome, and b) phylogenetic analysis of diversity panel including bread wheat (red) and SYN-DER (blue) genotypes, and c) genetic diversity visualization in diversity panel using 90K SNP array by principal component analysis.

**Figure 2:**
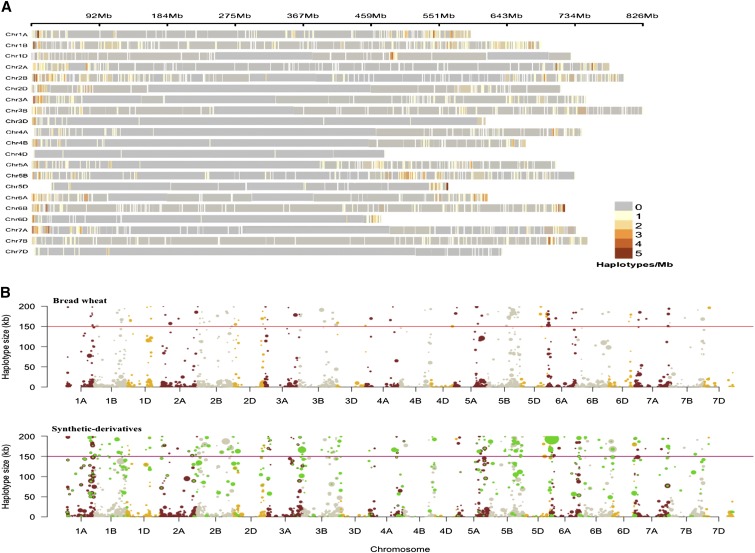
a) Haplotypes density along each of the wheat chromosomes. b) Distribution of haplotypes along wheat genome, where y-axis represent size of the haplotype block (kb) and size of the bubble represent number of SNPs/block. Green haplotype blocks are specific to the SYN-DER panel.

A total of 19,676 SNPs was retained after quality control (Table 1). SNP marker density on each wheat chromosome is visualized in Figure 1a. PIC value was highest on chromosomes 5A and 6A (0.35) and least on chromosomes 3D and 5D (0.19). Overall, PIC value was highest on the A genome (0.34) followed by the B genome (0.33) and D genome (0.31). In total, 2476 haplotypes were identified in SYN-DER with an averaged block size of 31.71 kb. Results from haplotype analysis followed similar trend to that of single SNP with most haplotypes identified on the B genome (1205) and chromosome 1B (245), while the least were identified on the D genome (328), particularly chromosome 4D (9). Haplotypes in BW and highlighted SYN-DER specific haplotypes are shown in Figure 2a and 2b. There was a significant difference between SYN-DER and BW for number of haplotypes and haplotype block size (kb); 23% of haplotypes were divergent in SYN-DER at chromosomal positions where BW did not generate any haplotype (Supplementary Table S2).

**Table 1:** Genome coverage, minor allele frequency (MAF) and polymorphic information contents (PIC) values on each chromosome in synthetic-derived diversity panel

	SNPs			Haplotypes			
Chr	Number	MAF	PIC	Number of haplotypes	SNPs/block	Average block size (kb)	Maximum block size (kb)
1A	1162	0.24	0.33	151	4	34.28	198.1
1B	1796	0.23	0.33	245	4	32.59	197.8
1D	422	0.31	0.34	77	4	31.77	195.7
2A	1197	0.29	0.33	157	3	29.04	199.6
2B	1940	0.25	0.34	247	4	28.55	199.8
2D	647	0.21	0.31	95	3	28.47	182.2
3A	988	0.24	0.33	129	4	42.11	199.6
3B	1360	0.24	0.33	163	4	33.09	197.25
3D	326	0.19	0.33	29	3	24.77	199.5
4A	831	0.25	0.34	102	4	21.98	198.4
4B	657	0.2	0.28	71	4	31.45	198.2
4D	143	0.21	0.3	9	3	24.36	192.8
5A	1173	0.26	0.35	135	4	40.84	199.7
5B	1819	0.24	0.33	202	4	51.58	198.8
5D	319	0.19	0.28	43	4	39.51	199.3
6A	1035	0.27	0.35	127	4	43.61	198.1
6B	1228	0.25	0.34	157	3	28.03	196.7
6D	229	0.26	0.33	48	3	25.14	181.02
7A	1080	0.24	0.33	142	4	29.32	194.84
7B	1057	0.25	0.34	120	4	26.78	190.7
7D	267	0.21	0.3	27	3	18.65	196.4
Group 1	3380	0.26	0.33	473	4	32.88	197.2
Group 2	3784	0.25	0.33	499	4	28.69	193.8
Group 3	2674	0.22	0.33	321	4	33.32	198.8
Group 4	1631	0.22	0.31	182	4	25.93	196.4
Group 5	3311	0.23	0.32	380	4	43.98	199.2
Group 6	2492	0.26	0.34	332	4	32.26	191.9
Group 7	2404	0.23	0.32	289	4	24.92	194.0
A	7466	0.26	0.34	943	4	34.45	199.7
B	9857	0.24	0.33	1205	4	33.15	199.1
D	2353	0.23	0.31	328	3	27.52	199.5
Total	19676	0.24	0.33	2476	4	31.71	195.9

PCA with high quality SNP markers clearly separated bread wheat cultivars from SYN-DER in various sub-clusters (Figure 1b). The first five principal components explained 39.7% of the total variability. Population structure based on PCA is presented in Figure 1c, where the first three PCs are presented as a 3D biplot. All the BW cultivars were in different clusters from SYN-DERs except those that were used in developing SYN-DERs.

**Figure 3:**
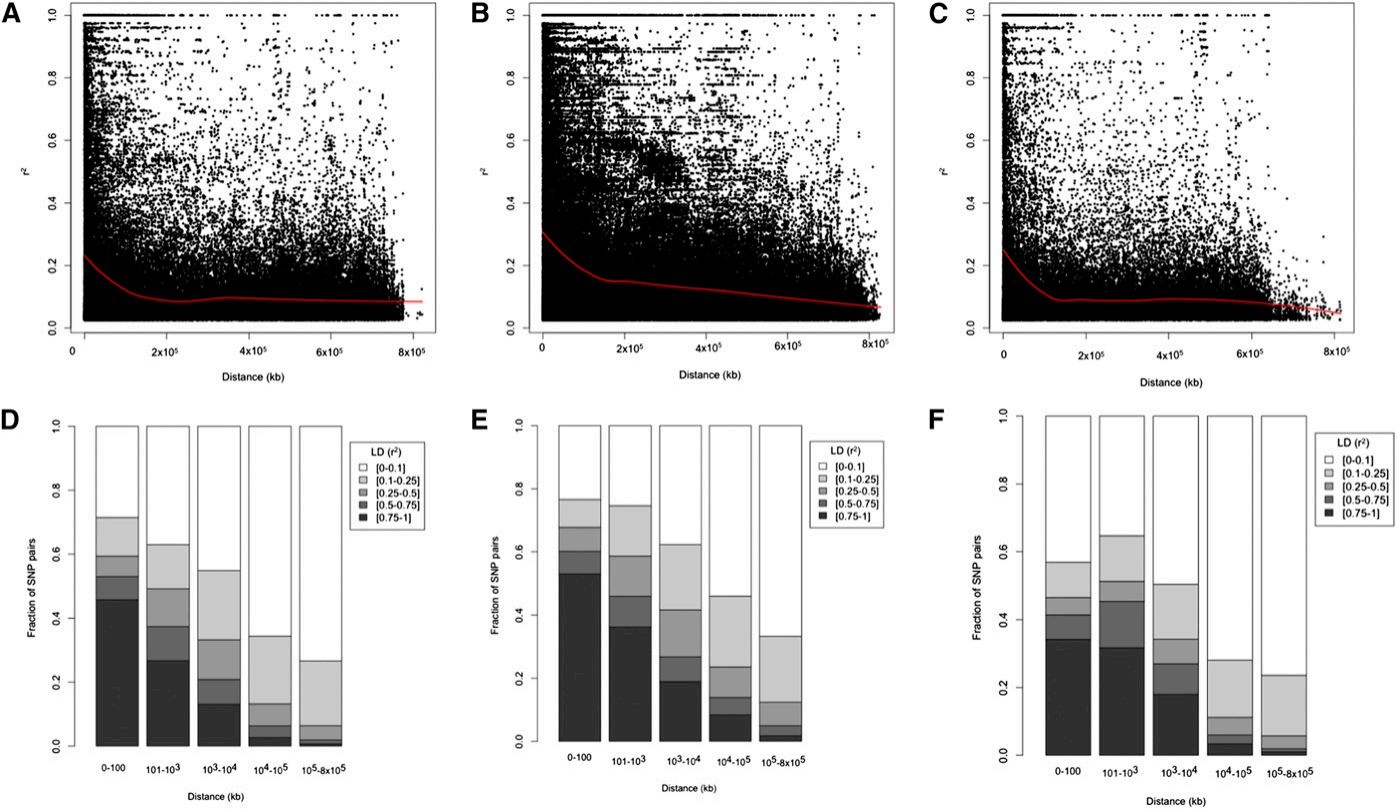
Linkage disequilibrium (LD) decay in the SYN-DER panel based SNP markers. The locally weighted polynomial regression-based (LOESS) representing decay of r^2^ along physical distance (kb) is illustrated for **a)** A-genome chromosomes, **b)** B-genome chromosomes, and **c)** D-genome chromosomes. Stacked bar plots of the LD statistic r^2^ as a function of physical distance (kb) between fraction of SNP pairs in SYN-DER for **d)** A-genome chromosomes, **e)** B-genome chromosomes, and **f)** D-genome chromosomes.

LD was estimated by *r^2^* at *P* ≤ 0.001 from all pairs of SNPs along each chromosome. Pairs of loci in significant LD on the sub-genome level was 45.55%, 47.47% and 35.85% on A, B and D genomes, respectively (Table 2). The average *r*^2^ of genome-wide LD was 0.22 in the A genome, 0.25 in the B genome and 0.36 in the D genome. The proportion of pairs of markers in complete LD was 2.97%, 4.43% and 4.75% on the A, B and D genomes, respectively. The extent and distribution of LD were graphically displayed in decay plots and bar plots between the fraction of SNP pairs in SYN-DER by plotting intrachromosomal r^2^ values for loci in significant LD at *P* ≤ 0.001 against the genetic distance in kb and a second-degree LOESS curve was fitted (Figure 3). The critical value for significance of r^2^ was estimated as 0.22 according to Breseghello and Sorrells (2006), and thus all values of r^2^ > 0.22 were estimated to be due to genetic linkage.

**Table 2:** Pairwise linkage disequilibrium statistics in synthetic-derived diversity panel classified into sub-genomes and various physical distance classes

Parameters	Genome	Classes			
		0-10Kb	11-100 Kb	100-1000Kb	>1000Kb
Total pairs	A	73072	259469	155402	167586
	B	105802	371339	209739	205547
	D	12833	47703	41567	91475
Significant pairs	A	33285	117226	63333	65116
	B	50225	169722	96122	85213
	D	4600	15855	14310	29608
Significant pairs (%)	A	45.55	45.18	40.75	38.86
	B	47.47	45.70	45.83	41.46
	D	35.84	33.24	34.43	32.37
Mean r^2^	A	0.22	0.18	0.16	0.14
	B	0.25	0.22	0.2	0.18
	D	0.36	0.30	0.23	0.20
Pairs in complete LD (%)	A	2.97	1.60	1.35	0.78
	B	4.43	2.82	2.46	1.60
	D	4.75	2.91	1.84	2.30
Pairs in r^2^ >0.5 (%)	A	7.26	5.44	3.76	2.98
	B	8.69	6.76	6.38	4.28
	D	12.07	9.01	5.94	6.76
Mean of r^2^ >0.5	A	0.82	0.78	0.8	0.78
	B	0.87	0.83	0.84	0.81
	D	0.84	0.82	0.8	0.82

### Phenotypic variations in diversity panel

Estimates of variance components showed significant differences among locations, treatments and genotypes for all traits. All the traits showed significant variations in WW and WL conditions in both SYN-DER and BW datasets (Figure S1). All the agronomic traits including GY, GS, PH, TP and TGW were significantly reduced by 19.4% (TGW) to 62.8% (GY) under WL conditions. However, CT, SOD, Sugar, Chl and Proline were significantly increased by 10.3% (CT) to 22.3% (SOD). In the WW environment, very high heritability (0.79 – 0.92) was observed for almost all physiological traits. In diversity panel, SYN-DER showed high performance on an average for all traits, for example GY in WL conditions was 16.7% higher in SYN-DER as compared to BW and can be visualized in violin distribution plots (Figure S1). In the WW environment, GY was positively correlated with SL (r = 0.29), TP (r = 0.61), GS (r = 0.40), and TGW (r = 0.74). Among biochemical traits, Chl was positively correlated with Sugar (r = 0.37), while proline was positively correlated with GS (r = 0.43) and SL (r = 0.28) (Figure S2). Other biochemical components had moderate to low correlation with grain yield and yield components. In the WL environment, GY was positively correlated with TGW (r = 0.66), GS (r = 0.72) and SL (r = 0.53), while SDW was negatively correlated with PH (r = -0.14) and Chl was negatively correlated with CTspike (r = -0.16) (Figure S2).

### GWAS for phenotypic traits based on SNPs

A total of 181 loci were associated with 15 phenotypes in SNP-GWAS, of which 66 were in WW conditions, 67 in WL and 62 were associated with the difference between WW and WL (DIFF) conditions (Table 3; Table S3). Of these 181 loci, 55 SNPs were located on the D genome, 22 of which were also identified as selective sweeps. An important chromosomal region on 1A between 512.2 to 574.3 Mb was associated with multiple physiological traits including RWC, CCI, SFW and SOD (Table S3). Nine SNPs were associated with GY_WW_, three with GY_WL_, and one with GY_DIFF_, of these four were located within selective sweep regions. Six SNPs were associated with GS_WW_, two with GS_WL_, and one with GS_DIFF_. Of these, two were distributed on chromosomes 1A and 6D that were under selection (Table S3). For TGW, an important yield component trait, three SNPs were associated with TGW_WW_, six with TGW_WL_, and 10 with TGW_DIFF_. Among these, six SNPs were under selective sweeps, two of which were located on the D genome (*i.e.*, chromosomes 3D and 7D). Three important loci were associated with phenotypic traits under both WW and WL conditions including SNP IWB20120 on chromosome 1A which was associated with sugar contents, IWB2303 on chromosome 1D linked to RWC and IWB8919 on chromosome 5B associated with TGW (Table 3).

**Table 3:** Marker-traits association based on SNP-GWAS identified in water-limited conditions

					*P* value*^d^*			
Trait*^a^*	Marker	Chr	MAF*^b^*	Position*^c^*	WW	WL	DIFF	Co-localization*^e^*
CCI	IWB9746	1A	0.22	551050619		1.52E-05		
CCI	IWB72768	3A	0.10	4352727		6.01E-05		7.78E-81
CCI	IWB21984	3B	0.09	39603457		9.05E-06		
CCI	IWB63113	3B	0.40	611176702		2.36E-08		4.67E-13
CCI	IWB6756	4A	0.30	24757405		1.58E-05		
CCI	IWB41634	5D	0.34	544614652		9.41E-09	2.52E-05	
CCI	IWB45289	7A	0.31	607556072		7.21E-05		*TaMoc1*
CCI	IWB27075	7D	0.12	627325333		2.64E-07		
Chl	IWB36330	1B	0.39	17892275		6.96E-04		6.31E-12
Chl	IWB58640	1D	0.17	206310986		5.88E-04		
Chl	IWA4651	4B	0.19	725661138		7.30E-04		
Chl	IWB28688	4D	0.50	2306721		3.94E-04		
Chl	IWB1744	5A	0.27	534083687		7.68E-04		1.36E-20
Chl	IWB7864	5B	0.34	2559480		3.97E-04		
CT	IWB37531	1A	0.07	9556706		2.11E-05		
GS	IWB51237	6B	0.26	291778439		7.74E-06	2.32E-06	
GS	IWB55521	7B	0.32	43880890		2.12E-05		
GY	IWB72112	2A	0.48	779818833		1.52E-05		
GY	IWB24186	4A	0.25	687548787		3.39E-05		*TaCwi-4A*
GY	IWB61578	5D	0.08	393057411		1.27E-06		1.95E-81
PH	IWB8266	5A	0.33	46232547		7.30E-05		
Proline	IWB61587	2A	0.27	5267865		4.06E-04	2.80E-04	3.63E-21
Proline	IWB48659	2B	0.29	664219887		6.77E-04		1.39E-18
Proline	IWA1156	7A	0.23	130613253		3.84E-04	4.46E-04	
RWC	IWB2303	1D	0.33	438369398	2.39E-09	2.84E-06		3.87E-17
RWC	IWB40605	2B	0.22	411517081		1.47E-08		
RWC	IWB31146	3B	0.17	409545629		5.49E-06		2.44E-37
RWC	IWB61562	3D	0.39	6955053		2.01E-05		
RWC	IWB50770	3D	0.28	311662828		4.88E-05		3.28E-19
RWC	IWB53305	5B	0.11	327790108		6.28E-08		1.06E-60
RWC	IWB13408	6A	0.07	542232293		3.94E-06		
RWC	IWB43804	6D	0.25	7104862		4.90E-05		
RWC	IWB43363	7A	0.07	705688072		2.99E-06		Hap-7A-RWC1,1.53E-108
SDW	IWB75332	1D	0.21	325672368		2.89E-05		1.97E-27
SDW	IWB33249	5D	0.42	4608766		6.47E-05		6.26E-07
SDW	IWB15529	6D	0.30	50743512		6.41E-05		3.05E-17
SFW	IWB11543	1A	0.37	512878328		6.18E-05		
SFW	IWA5594	3D	0.12	782836300		6.64E-05		
SFW	IWB63863	5A	0.09	685991984		6.84E-06		3.44E-74; AWN
SFW	IWB59042	5B	0.10	220817473		2.08E-06		
SFW	IWB18780	6D	0.40	143644031		7.58E-07		3.73E-12
SFW	IWB67174	7D	0.20	3924400		8.68E-06		
SL	IWB48031	1B	0.34	49886407		1.98E-04		
SL	IWA4073	1D	0.27	8726971		8.93E-04		8.14E-21
SL	IWB48188	2A	0.26	776290034		1.81E-04		
SL	IWB61861	2B	0.27	69014963		6.41E-04		
SL	IWB55762	6A	0.10	615780928		3.14E-04		
SL	IWB14155	6B	0.14	715704473		7.52E-04		
SL	IWA1949	7A	0.31	53051117		8.78E-04		
SL	IWB6450	7A	0.31	712309084		7.54E-04		6.65E-17
SOD	IWB29553	3D	0.09	496676602		8.98E-05		
SOD	IWB4492	5D	0.07	394102098		5.52E-05		
Sugar	IWB20120	1A	0.07	224826985	6.45E-05	4.54E-04		
Sugar	IWB53805	3A	0.43	513608135		3.79E-04	2.60E-04	
Sugar	IWB56874	3D	0.46	5889027		3.75E-04		
Sugar	IWB23415	5D	0.20	549864711		7.83E-04		
Sugar	IWB70872	6A	0.17	545828799		2.93E-04		
TGW	IWB65286	2A	0.18	553276150		4.03E-04		*TaCwi-A1*
TGW	IWB6655	3A	0.10	657945858		3.30E-04		7.17E-86
TGW	IWB16311	3D	0.25	48365010		6.51E-04		
TGW	IWB8919	5B	0.09	576923927	7.28E-04	1.90E-04		*Vrn-B1*
TGW	IWB58464	6A	0.09	38453171		6.28E-04		3.03E-96
TGW	IWB15372	7D	0.25	559459585		3.34E-04		Hap-7D-TGW1
TP	IWB61466	1B	0.34	520899317		5.04E-04		8.57E-16
TP	IWA3583	2B	0.12	535137547		1.59E-04		6.75E-66
TP	IWB73711	3A	0.22	57012959		3.09E-04		
TP	IWB10457	4A	0.24	690845337		5.98E-04		4.70E-26
TP	IWB6762	5A	0.13	535138119		3.50E-04		*Vrn-A1*

a Chl: chlorophyll contents (mg/g); CCI: chlorophyll content index; CT: Canopy temperature (°C); GS: grains per spike; GY: grain yield (g m^-2^); PH: plant height (cm); Proline (µmol/g); RWC: relative water contents (%); SDW: shoot dry weight (g); SFW: shoot fresh weight (g); Sugar (µg/g); TGW: thousand grain weight (g); TP: tillers per plant.

b MAF: Minor allele frequency.

c Position of SNP based on IWGSC Ref sequence version 1.0 (https://urgi.versailles.inra.fr/blast_iwgsc/blast.php)

d P-value significance for marker-trait associations in well-watered (WW), water-limited (WL) and difference between well-watered and water-limited (DIFF) conditions.

e Co-localization of marker-trait associations with divergent selective sweeps (in case of p-value) and/or functional genes.

### GWAS for phenotypic traits based on haplotypes

In haplotype-GWAS, 127 haplotypes were associated with phenotypes under different water regimes. Of these, 71 were identified in WW, 43 in WL and 20 in ‘Diff’ (Table 4; Table S4). Co-localization of loci under divergent selection and haplotype-GWAS identified 23 loci within the regions under selection in SYN-DERs (Table 4 and Supplementary Table S4). Only eight loci were common under both SNP-GWAS and haplotype-GWAS, including Hap-2B-RWC2, Hap-2B-CCI, Hap-3D-SFW1, Hap-4A-PH1, Hap-5A-GY2, Hap-7A-RWC1, Hap-7B-TGW3, and Hap-7D-TGW1. Due to the greater diversity of the D-genome in SYN-DER, 21 haplotypes were identified on D genome, of which seven haplotypes were under divergent selection. Thirteen haplotypes were associated with GY_WW_, four with GY_WL_, and one with GY_DIFF_, one of which was in the region of selective sweeps. Similarly, two haplotypes were associated with GS in WL. Nineteen haplotypes were identified for TGW_WW_ and one for TGW_WL_; four of these were under selective sweeps and two were located on the D genome (*i.e.*, chromosome 7D). Three of the TGW associated haplotypes were under divergent selection, while two haplotypes were closely linked to vernalization (*Vrn-A1* and *Vrn-B1* genes) response on chromosomes 5A and 5B.

**Table 4:** Marker-traits associations based haplotype-GWAS identified in water-limited conditions

					*P*-values*^c^*			
Trait*^a^*	Haplotype block	Chr	Position*^b^*	Haplo-group	WW	WL	Diff	Co-localization*^d^*
Chl	Hap1B-Chl-1	1B	4320839	GATACT		7.93E-06		
Chl	Hap3B-Chl-1	3B	480341620	TT		2.05E-06		
Chl	Hap4A-Chl-1	4A	12399327	CC		8.00E-06		
Chl	Hap4A-Chl-2	4A	726215255	GA		1.64E-07		
Chl	Hap4A-Chl-2	4A	726215255	AG		2.48E-07		
Chl	Hap4B-Chl-1	4B	651800378	TCGGCCC		1.61E-08		
Chl	Hap6A-Chl-1	6A	4427440	CA		5.26E-06		
CT	Hap4A-CT-1	4A	614870817	CC		8.91E-06		
GS	Hap1B-GS-1	1B	548536105	TTTTCTGCCAATTGGCGTGTCAG		2.68E-06		
GS	Hap4A-GS-1	4A	631922510	TTAT		9.28E-06		
GY	Hap1A-GY-1	1A	12306177	CT		2.63E-06		1.16E-46
GY	Hap1B-GY-1	1B	20589424	ACG		6.17E-06		
GY	Hap5B-GY-1	5B	10888795	CC		5.33E-06		
GY	Hap7B-GY-1	7B	3148623	AC		5.74E-06		
PH	Hap1B-PH-1	1B	15166232	GCA		3.04E-06		
PH	Hap1B-PH-1	1B	15166232	AAG		3.04E-06		
PH	Hap2A-PH-1	2A	118446554	TC		4.54E-06		*Sus2-2A*
PH	Hap2A-PH-2	2A	319042351	CC		8.94E-06		
PH	Hap2A-PH-3	2A	712706867	TGC		6.67E-09		1.56E-25
PH	Hap2A-PH-3	2A	712706867	CAT		1.88E-08		1.56E-25
PH	Hap2B-PH-3	2B	683003143	TGCTTTAT		4.41E-08		
PH	Hap2D-PH-1	2D	9826098	GG		9.75E-09		
PH	Hap2D-PH-3	2D	572074690	CTTTC		3.09E-08		
PH	Hap2D-PH-3	2D	572074690	TCGCT		3.09E-08		
PH	Hap3B-PH-1	3B	24940169	TAG		8.74E-06		
PH	Hap4A-PH-1	4A	732511707	GACAT		4.22E-07		1.74E-31
PH	Hap5A-PH-1	5A	679489793	GAGAAA		2.15E-06		AWN
PH	Hap5B-PH-1	5B	580085162	GG		8.42E-06		
PH	Hap6B-PH-1	6B	15781175	CCG		6.64E-06		
PH	Hap6B-PH-2	6B	709531181	GTT		8.41E-06		
Proline	Hap4D-Proline-1	4D	42538561	GT		1.60E-06	4.70E-07	
RWC	Hap2A-RWC-1	2A	41232496	ACTCCG		5.10E-06		*Ppd-A1*
RWC	Hap2B-RWC-2	2B	643682900	CGTGGGG	7.48E-06	4.94E-06		
RWC	Hap3B-RWC-1	3B	40184024	TGTAGGGC		3.01E-06		
RWC	Hap7A-RWC-1	7A	701493027	GACTAAC		8.85E-06		
SDW	Hap2D-SDW-1	2D	64190307	AG		7.56E-06	1.19E-07	
SFW	Hap2A-SFW-1	2A	709836654	ATAG		8.35E-06		
SFW	Hap2D-SFW-1	2D	571077641	GCAATTAGT		7.27E-07		2.19E-21
SFW	Hap3A-SFW-1	3A	666229575	CG		1.81E-06		
SFW	Hap3A-SFW-1	3A	666229575	TA		1.88E-06		
Sugar	Hap4B-Sugar-1	4B	535049503	TTGCGAAA		4.23E-06		
Sugar	Hap4B-Sugar-1	4B	535049503	CCAAAGGG		4.23E-06		
TGW	Hap5B-TGW-2	5B	603881835	ATTCTCGTT		4.18E-06		

a Chl: chlorophyll contents (mg/g); CCI: chlorophyll content index; CT: Canopy temperature (°C); GS: grains per spike; GY: grain yield (g m^-2^); PH: plant height (cm); Proline (µmol/g); RWC: relative water contents (%); SDW: shoot dry weight (g); SFW: shoot fresh weight (g); Sugar (µg/g); TGW: thousand grain weight (g).

b Position of SNP based on IWGSC Ref sequence version 1.0 (https://urgi.versailles.inra.fr/blast_iwgsc/blast.php)

c P-value significance for marker-trait associations in well-watered (WW), water-limited (WL) and difference between well-watered and water-limited (DIFF) conditions.

d Co-localization of marker-trait associations with divergent selective sweeps (the numeric values are the p-value of selection sweep) and functional genes.

### Allelic effects of SNPs and haplotypes on phenotypes

**Figure 4:**
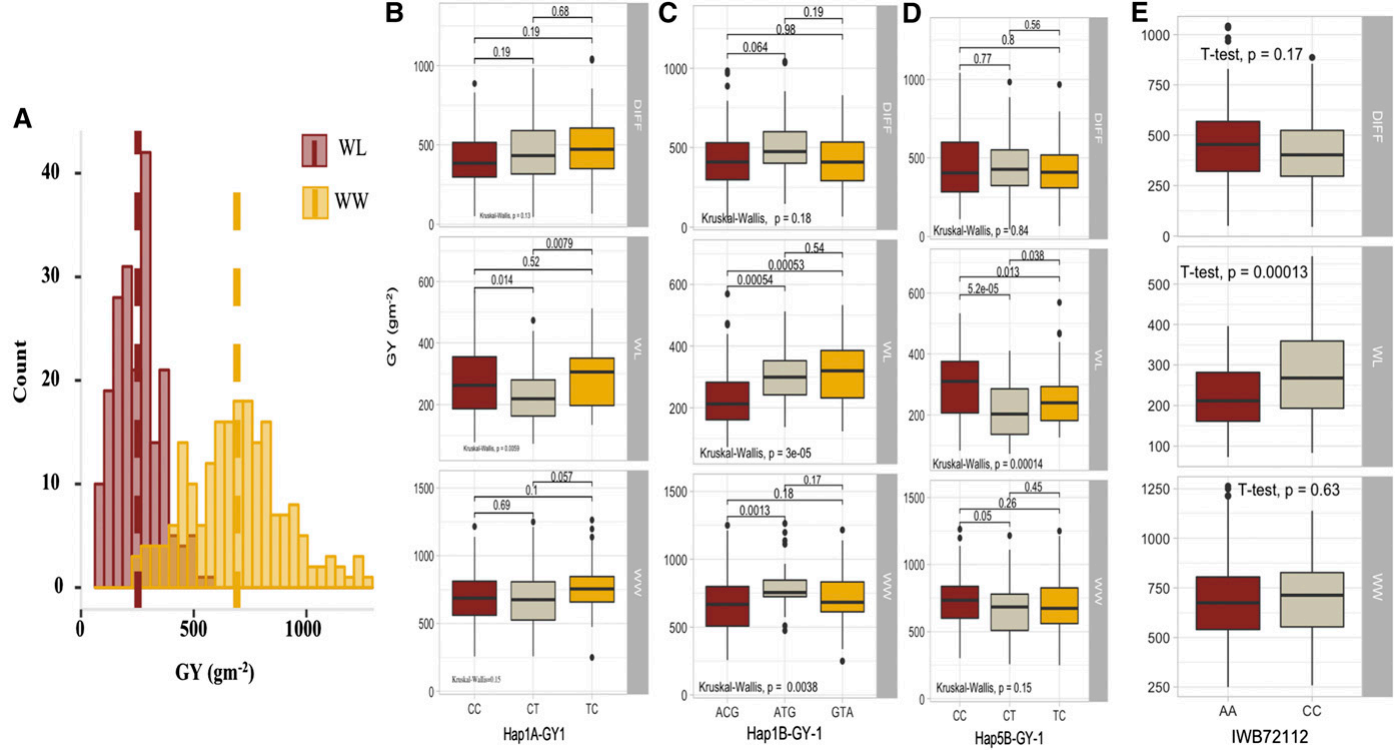
Histogram for grain yield and boxplot for SNPs and haplotypes associated with grain yield in different water regimes. **a)** Histogram for grain yield under well-watered (WW) and water-limited (WL) conditions. Allelic effects of Hap1A-GY1 **b)**, Hap1B-GY1 **(c)**, Hap5B-GY1 **(d)** and SNP IWB72112 **(e)** on GY across three water regimes (all information is annotated).

**Figure 5:**
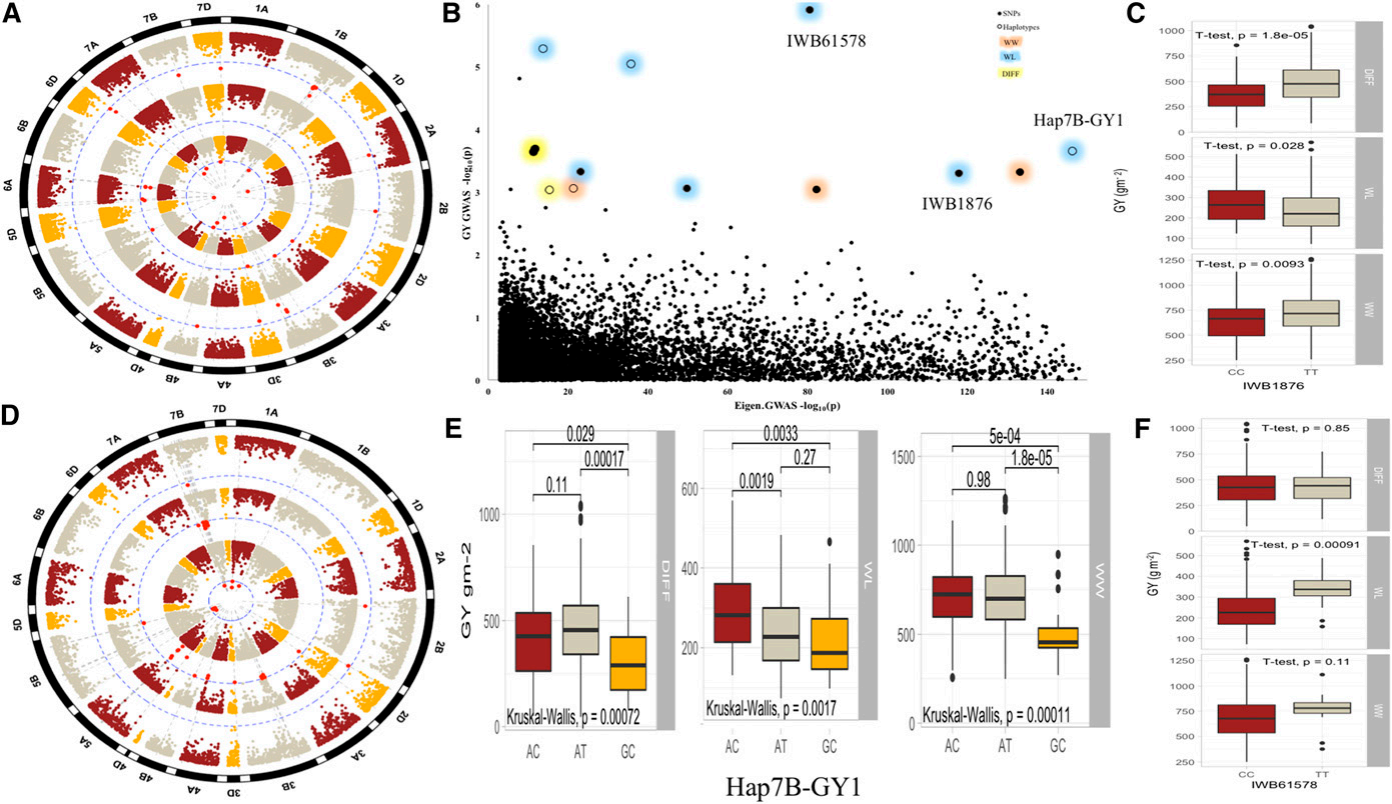
a) Manhattan plots for SNP-GWAS for GY in WL (inner-most), WW (middle) and DIFF (outer-most) conditions, b) Biplot showing co-localization of GY-GWAS and EigenGWAS highlighting the loci under divergent selections, c) Allelic effects of SNP (IWB1876) on GY across three water regimes, d) Manhattan plots for haplotype-GWAS for GY in WL (inner-most), WW (middle) and DIFF (outer-most) conditions, e) Allelic effects of haplotype (Hap7B-GY1) on GY across three water regimes, f) Allelic effects of SNP (IWB61578) on GY across three water regimes.

**Figure 6:**
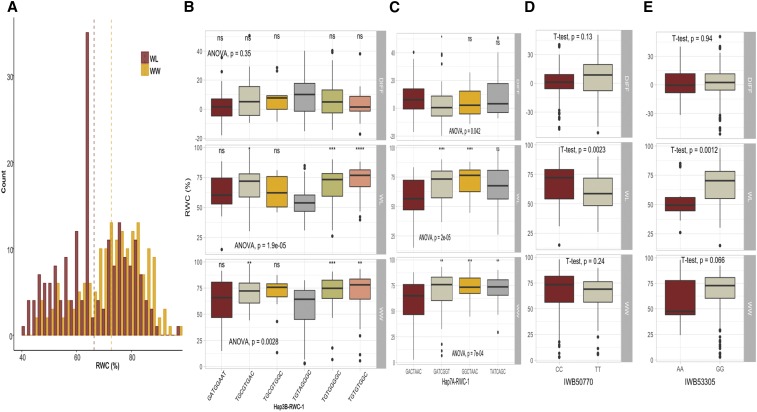
Histogram for relative water contents (RWC %) and boxplot for SNPs and haplotypes associated with RWC in different water regimes. **a)** Histogram for RWC under well-watered (WW) and water-limited (WL) conditions. Allelic effects of Hap3B-RWC1 **b)**, Hap7A-RWC1 **c)**, SNP (IWB50770) **d),** SNP (IWB53305) **e)** on RWC across three water regimes (all information is annotated).

**Figure 7:**
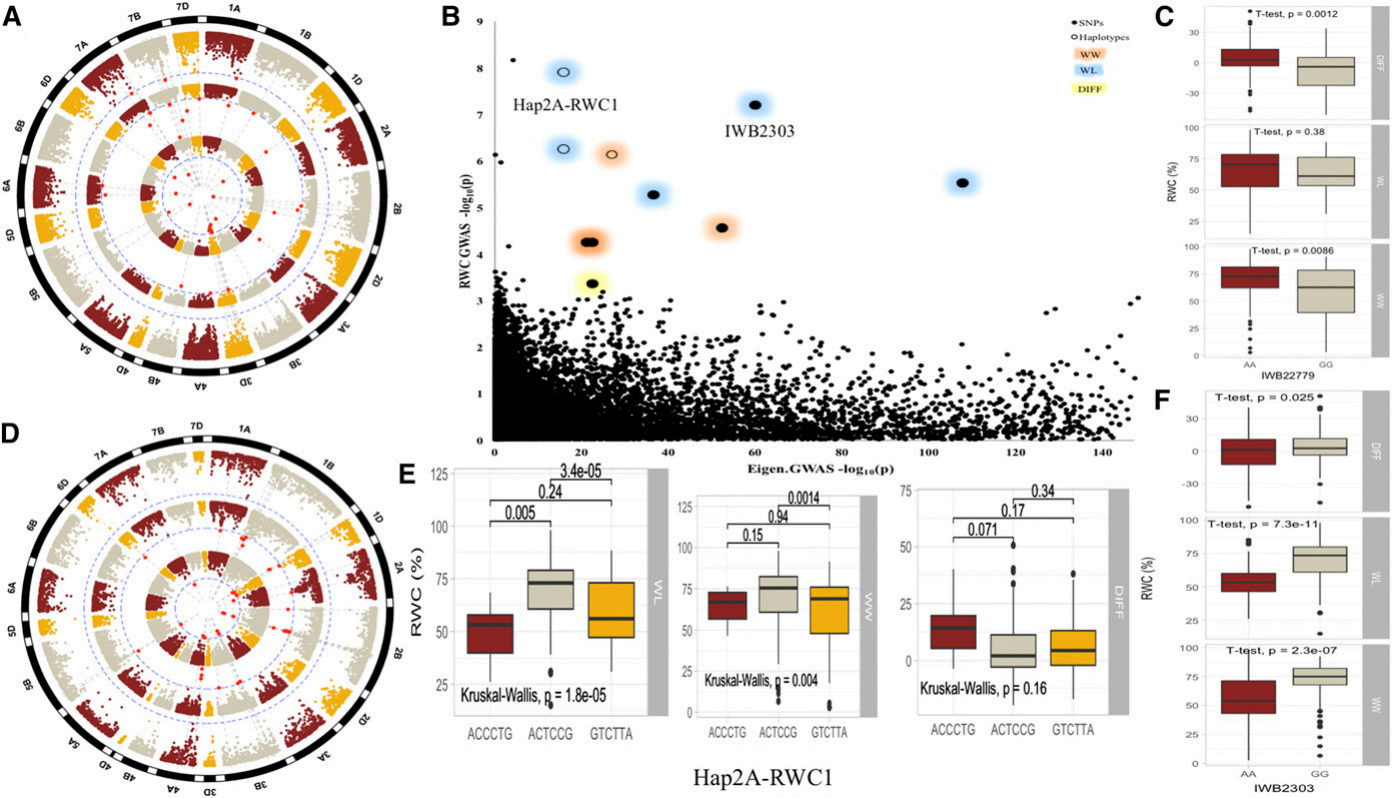
**a)** Manhattan plots for SNP-GWAS for relative water contents (%) in WL (inner-most), WW (middle) and DIFF (outer-most) conditions, **b)** Biplot showing co-localization of RWC-GWAS and EigenGWAS highlighting the loci under divergent selections, **c)** Allelic effects of SNP (IWB22779) on RWC across three water regimes, **d)** Manhattan plots for haplotype-GWAS for RWC in WL (inner-most), WW (middle) and DIFF (outer-most) conditions, **e)** Allelic effects of haplotype (Hap2A-RWC1) on RWC across three water regimes, **f)** Allelic effects of SNP (IWB2303) on RWC across three water regimes.

SNPs and haplotypes associated with two important phenotypic traits; GY and RWC were used to show the allelic effects in each of the WW, WL, and ‘DIFF’ environments (Figure 4 - Figure 7). Three SNPs (Figure 4e and Figure 5c, f) and four haplotype blocks (Figure 4b,c,d) associated with GY_WL_ were assessed for their allelic effects on phenotypes in all water regimes. Among these SNPs, only IWB1876 on chromosome 4A was associated with GY_WL_, GY_WW_ and GY_DIFF_, while another two SNPs; IWB72112 (2A) and IWB61578 (5D) were only associated with GY_WL_. These SNPs gave a yield advantage of 46.2, 26.5 and 28.2 gm^-2^ in water-limited conditions. Each of the three haplotype blocks consisted of three haplo-groups and favored haplo-groups significantly increased GY_WL_ by 39.7 to 87.2 gm-^2^. Similarly, the favored SNP alleles gave an advantage of 2.9–6.3% for RWC_WL_. Three haplotype blocks; Hap2A-RWC1, Hap3B-RWC1 and Hap7A-RWC1 were associated with RWC_WL_ and consisted of 3, 6, and 4 haplo-groups, respectively (Figure 6b,c,d). The favored haplo-groups in each haplotype block were associated with 3.4–8.7% increases in RWC_WL_.

### Divergent selections and their co-localization With drought QTL in SYN-DER wheats

**Figure 8:**
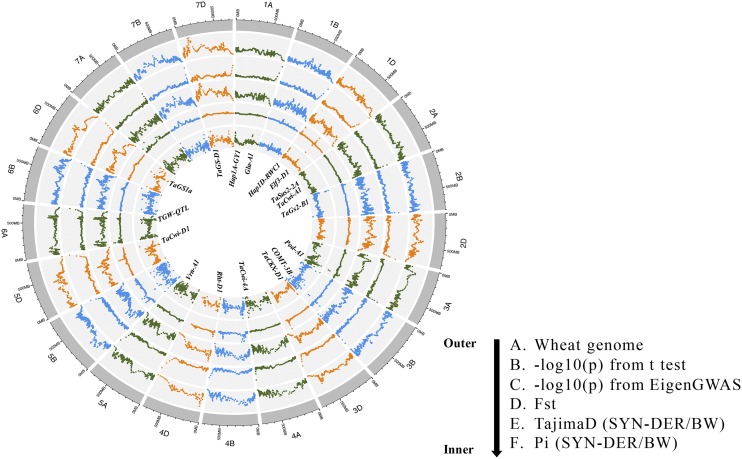
Circos plot of five analytical procedures (legends are given above) used to identify selection loci in SYN-DER and modern bread wheat panels and then highlighting ‘divergent selections’ in SYN-DER.

Genome-wide divergent selection was investigated in SYN-DER by comparing allele frequencies in the two panels. Five different approaches were used to provide complementary information (Figure 8; Table S5). The Student’s *t*-test was used to identify significantly different allele frequencies (Table S5; Figure 5). *Fst* was calculated with a sliding window approach and only the top 5% of *Fst* values were regarded as selective loci. This was further validated by a new EigenGWAS approach where eigenvectors were used as phenotypes for GWAS. Tajima’s D and π were calculated in each of the subpopulations. All the results were analyzed comparatively to identify genomic regions consistently under divergent selection in SYN-DER. In total, 291 SNPs representing selective sweep were narrowed down to 89 loci based on the LD (r^2^ > 0.7). These 89 loci or divergent selections were present on all 21 chromosomes (Table S5). The number of loci under divergent selection differed among the three genomes, and the most loci were located on the D-genome (32), followed by the B- (29) and A-genomes (28). Similarly, EigenGWAS was plotted against SNP-GWAS and haplotype-GWAS to highlight GY and RWC associated loci under divergent selection (Figure 5b and Figure 7b, respectively). Among these loci, 31 were regarded as ‘hard sweeps’ where contrasting alleles were fixed (MAF > 0.9) in both subsets or where the –log(P) value for EigenGWAS was among the top 5% (Table S5). Based on the extent of LD in the SYN-DER panel, 20 loci were within the close proximity of functional genes such as *Elf3-D1* and *Vrn-D3* for flowering time, *TaCwi-A1*, *TaCKX-D1*, *TaSus1-7A*, and *TaGS-D1* for grain size and weight, and *1fehw3*, *TaMoc1* and *SST-A1* for sugar metabolism and transport (see Table S5 for complete list of genes).

## Discussion

### Genetic diversity in SYN-DER

Crop wild relatives provide enhanced diversity and new favorable genes and gene recombinations that potentially increase additive variation for traits of breeding interest. The goat grass (*Ae. tauschii*) reference genome sequence has recently been published (Luo *et al.*, 2017; Luo *et al.*, 2013; Zhao *et al.*, 2017). This sequence is an important tool for better understanding of wheat biology, adaptation and productivity. In parallel, *Ae. tauschii* diversity has been globally exploited in breeding through the use of synthetic hexaploid wheats (Börner *et al.*, 2015; Ogbonnaya *et al.*, 2013). The current study showed that SYN-DER wheats have different patterns of genetic diversity and population structure compared to modern bread wheat as revealed by PCA, phylogenetic tree and haplotype blocks (Figure 1 and 3). Since these SYN-DER have been selected in the field for agronomic superiority, it is possible that major proportions of the bread wheat genome have been retained because empirical selection can lead to a differential loss of genetic diversity in targeted genomic regions of agronomic importance. This in return enhances the diversity of neutral genes through promotion of gene flow, recombination and exchanges between diverse populations (Olsen and Wendel 2013). There is potential ascertainment bias in the 90K SNP array because the SNP array was developed based on transcriptome sequencing of few bread wheat cultivars (Wang *et al.*, 2014) and variation in promoter regions and introns where much of the variation is present were not surveyed (Zheng *et al.*, 2014). Despite these limitations, we were able to identify differential patterns of diversity and 89 loci were under divergent selection in SYN-DERs.

### Important candidate regions identified by GWAS for agronomic and physiological traits in SYN-DER

GWAS was performed on 15 agronomic and physiological traits under two water regimes (WW and WL) and their difference (DIFF) leading to 45 phenotypic datasets in total. We further calculated LD between all SNPs associated with phenotypes and excluded those SNPs with *r^2^* > 0.8 and finally 181 MTAs (66 in WW, 68 in WL, and 63 in DIFF) are presented for 45 phenotype datasets. SNP-GWAS identified a relatively high number of MTAs (181) compared to haplotype-GWAS (128). We used physical positions of SNPs based on Chinese Spring reference genome sequence version 1.0 (https://urgi.versailles.inra.fr/download/iwgsc/IWGSC_RefSeq_Assemblies/v1.0/), while all the previous studies used genetic maps based on linkage mapping population. Therefore, it is hard to compare the results with previous findings. Acuña-Galindo *et al.* (2015) previously used a meta-analysis approach and developed consensus QTL positions from various mapping experiments. As the reference genome sequences of bread wheat and some progenitors are available (Avni *et al.*, 2017; Luo *et al.*, 2017; Zhao *et al.*, 2017), there is urgent need to translate QTL location information to a position in the wheat genome. We found very few common MTAs between WW and WL (3), WW and DIFF (9) and WL and DIFF (5). Such a phenomenon has been reported in most of the abiotic stress experiments where QTL under c ontrol conditions do not significantly overlap with QTL under stress conditions probably due to different evolutionary trajectories (Makumburage and Stapleton 2011; Ogbonnaya *et al.*, 2017).

A yield MTA for GY_WL_ on chromosome 5D (393.05 Mb) is a novel MTA because it is under strong divergent selection (-log(p)=81.9) and the favorable allele gave a yield advantage of 76.2 gm^-2^. This MTA was also associated with SOD_WL_. Two other MTAs for GY_WL_ on chromosomes 2A and 4A are likely to be MQTL13 and MQTL32 (Acuña-Galindo *et al.*, 2015) based on genetic positions of associated SNPs on the wheat consensus genetic map (Quraishi *et al.*, 2017). A TGW_WL_ MTA on chromosome 2A (553.2 Mb) is within the vicinity of grain weight gene *TaCwi-A1* (Ma *et al.*, 2012). Based on the association with multiple phenotypes, we identified three genomic important regions such as chromosome 1D at 438.3 Mb that was associated with physiological traits RWC, SFW, and SDW. RWC in leaves is a sensitive parameter for estimating plant water balance (Clavel *et al.*, 2005), and also shows a positive relationship with yield in cereals (Merah 2001). The second important region on chromosome 3D (5.1-48.3 Mb) is associated with Sugar, RWC, and TGW which are important parameters in drought adaptability. This genomic region was not under divergent selection, so it is likely that it was retained from bread wheat parents of SYN-DER. A third important genomic region on chromosome 6A (542.7-615.3 Mb) was associated with RWC, Sugar and SL. Based on genetic positions, these two regions are likely to be the MQTL29 and MQTL49 based on the meta-QTL map (Acuña-Galindo *et al.*, 2015). Two drought tolerance related genes *TaCwi-4A* and *TaMoc1* (7A) were co-localized with GY_WL_ and CCI_WL_ MTAs. Zhang *et al.* (2015) reported that *TaMoc1* is an important gene associated with yield-related traits and is involved in regulating axillary meristem initiation and growth, while *TaCwi-4A* is an important drought related gene only expressed in anthers and is involved in pollen sterility under drought stress. The association of these genes directly with GY_WL_ in SYN-DER indicated the potential usefulness of this MTA because the minor allele frequency is higher (0.25), while the *TaCwi-4A* favorable allele is highly fixed during modern wheat breeding (Jiang *et al.*, 2015).

### Haplotype-GWAS, a powerful complement With SNP-GWAS

Consistent with reports from other studies, haplotype-GWAS proved to be an effective strategy to increase the resolution of GWAS experiments. However, this strategy is rarely implemented in wheat, even though it has shown promise in Brassica (Wu *et al.*, 2016), barley (Lorenz *et al.*, 2010) and other crop species (Qian *et al.*, 2017). Our results were in agreement with Hao *et al.* (2017) that haplotype-GWAS was able to identify MTAs where individual SNPs were ineffective. This was because haplotypes containing a group of closely linked SNP markers can increase the level of polymorphisms and overcome the drawback of using single SNP markers by creating more combinations (haplotypes) (Supplementary Table S3). This can increase the power and the effectiveness of haplotype-based association (Hamblin and Jannink 2011; Lorenz *et al.*, 2010). Several haplotypes associated with phenotypes in our study were not identified by SNP-GWAS and it is likely due to the fact that patterns of LD in the population and the marker density along with the genetic architecture of the trait affect the detection of haplotypes associated with phenotypes (Lorenz *et al.* 2010). Moreover, the information content of haplotypes is dependent on the particular mutational and recombinational history of the QTL and nearby markers which could be unlike the distribution of single SNPs. Therefore, it was recommend to routinely use both single SNP and haplotype markers for GWAS to take advantage of the full information content of the genotype data.

We identified three haplotypes associated with GY_WL_ and each haplotype had three haplo-groups (Figure 4). The favored haplotypes significantly increased GY_WL_ by 39.7 to 87.2 gm^-2^. This is important because none of the single SNPs were associated with GY_WL_ in these genomic regions. Similarly, Hap-7B-GY-1 was associated with grain yield across three water treatments *i.e.*, WW, WL and DIFF, while only two haplo-groups (AC and GC) of this haplotypes block showed significant differences for GY and a third haplo-group ‘AT’ remained non-significant. In haplotype block Hap-1B-GY-1, there was no significant difference between GY_WL_ of two haplo-groups ‘ATG’ and ‘GTA’, while the ‘ACG’ variant had a significantly different effect on GY (Figure 4). Haplotype-GWAS identified several genomic regions closely linked to functional genes such as a haplotype on chromosome 2B (643.6-690.1 Mb) that was associated with multiple physiological traits RWC, SFW and PH and was linked to glutamine synthase2 gene, *TaGS2-B1* (Li *et al.*, 2011). Previously, haplotypes at *TaGS2-B1* were associated with nitrogen use efficiency and several agronomic traits including shoot and root dry weight (Li *et al.*, 2011), which corroborates results from this study. Similarly, haplotypes on chromosome 5A (504.6 Mb) associated with TGW and GY are within the same region of the *Vrn-A1* gene. On chromosome 5B (603.8 Mb), the haplotype associated with TGW is within the proximity of *Vrn-B1*. These two genes are homeologous to VERNELIZATION1 (*Vrn1*) gene, a key regulator of flowering time in cereals that have pleiotropic effects on agronomic traits like plant height and root traits (Chen *et al.*, 2015; Voss-Fels *et al.*, 2017). Two closely linked haplotypes on chromosome 2A (31.9-41.2 Mb) were linked to functional genes *Ppd-A1* (Beales *et al.*, 2007) and *Sus2-2A* (Hou *et al.*, 2014), which modulate the flowering time and yield related traits, respectively. Most of the synthetic hexaploid wheats have the GS-105 type 1,117 bp deletion in *Ppd-A1* gene (M. Khalid *et al.*, unpublished data), which is the *Ppd-A1a* photoperiod insensitive allele in durum wheat. This allele is different from *Ppd-A1a* allele in bread wheat and has higher transcript levels (Wilhelm *et al.*, 2009). In conclusion, the simultaneous use of SNP-GWAS and haplotype-GWAS dissected the complex quantitative traits at higher resolution and several genomic regions harboring functional and candidate genes of agronomic importance were identified.

### Divergent selections associated with phenotypes and future breeding strategies

Although traditional linkage mapping and association mapping are effective for identifying large-effect trait loci, they are limited to phenotypes used in analyses and may fail to detect a large portion of the genetic changes associated with plant domestication and improvement (Morrell *et al.*, 2011). During the development of adapted lines, selection imposed by humans favored alleles of traits valuable for agriculture. When selection increases the frequency of beneficial alleles in a population, it impacts the standing variation of surrounding genomic regions resulting in reduced diversity, extended linkage disequilibrium, or strong inter-population allele frequency differentiation. To detect these local patterns of variation, also referred here to as ‘divergent selective sweeps’, we first investigated the genome-wide divergent selections in SYN-DER panel using various complementary analytical approaches (Supplementary Table S4). However, only some of these selection loci could be associated with phenotypes. In a second step, we compared the SNPs in selection loci to those identified in SNP-GWAS and haplotype-GWAS to narrow down the selection loci associated with phenotypes of significant agronomic value. This approach resulted in the identification of 53 SNPs (Table 3) and 23 haplotypes (Table 4) of agronomic importance that appeared to be loci under selection. We have shown selection loci as EigenGWAS as –log10(p) associated with GY and RWC (Figure 5b and 7b). These loci could provide novel adaptability and agronomic superiority in drought stressed environments.

As the narrow genetic base of the D-genome in bread wheat is widely acknowledged, divergent haplotypes in the D-genome in SYN-DER could be a mean to introduce new genetic diversity for traits of breeding interest. The co-localization of these haplotypes with functional genes strongly supported the idea that conventional haplotypes strongly fixed during domestication or modern breeding have been replaced by new haplotypes from *Ae. tauschii* through synthetic wheats. For example, the close proximity of these haplotypes with functional genes like *TaElf3-D1* (1D at 468.7 Mb) and *Vrn-D3* (7D at 70.8 Mb) for earliness *per se* (Eps) and flowering time (Wang *et al.*, 2009; Zikhali *et al.*, 2016), *TaCKX6-D1* (3D at 137.4 Mb), *TaCwi-D1* (5D at 557.3 Mb) and *TaGS-D1* (7D at 6.7 Mb) (Jiang *et al.*, 2015; Zhang *et al.*, 2012; Zhang *et al.*, 2014) for grain size and weight indicated that novel genetic introgressions from synthetic hexaploid wheats may have been selected.

The benefit of using synthetic wheats or other crop-wild introgressions is that new useful haplotypes could be generated due to the differential recombination rates and patterns (Rasheed *et al.*, 2018; Wingen *et al.*, 2017). Such new favorable haplotypes contribute to additive genetic variation for complex quantitative traits, which can replace the conventional haplotypes exhaustively utilized during modern wheat breeding; thus improving the rate of genetic gains. The breeding strategies such as genomic selection can be applied following the identification of molecular markers linked to those haplotypes. For example, Spindel *et al.* (2016) identified SNPs associated with agronomic traits and fitted these markers as fixed effects in a rrBLUP model (referred to as a GS + *de novo* GWAS model). They showed that this new model outperformed six other prediction models for various traits. Furthermore, the prediction accuracies of extended GS models outperformed classical models using phenotype data from dry seasons, implying that this approach is particularly suitable for the improvement of drought-resilience in future crop cultivars. Because of their increased information content compared to bi-allelic SNP markers, fitting haplotypes with statistically significant trait associations to phenotypes as fixed effects in GS models could further improve prediction accuracies (Voss-Fels *et al.*, 2017). The use of haplotype-assisted GS should more accurately depict the complex relationships between genotypic information and phenotypes than single SNPs alone; hence, this approach could ultimately help to further increase selection gain per unit of time.

Our analytical approaches successfully identified the divergent selections in SYN-DER, dissected the complex architecture of quantitative traits under drought stress and identified the favorable SNP and haplotypes underpinning traits of breeding interest through crop-wild introgressions.

## References

[bib1] Acuña-Galindo M. A. Mason R. E. Subrahmanyam N. K. Hays D., 2015 Meta-analysis of wheat QTL regions associated with adaptation to drought and heat stress. Crop Sci. 55: 477–492. 10.2135/cropsci2013.11.0793

[bib2] Afzal F. Reddy B. Gul A. Khalid M. Subhani A., 2017 Physiological, biochemical and agronomic traits associated with drought tolerance in a synthetic-derived wheat diversity panel. Crop Pasture Sci. 68: 213–224. 10.1071/CP16367

[bib3] Akhunov E. Nicolet C. Dvorak J., 2009 Single nucleotide polymorphism genotyping in polyploid wheat with the Illumina GoldenGate assay. Theor. Appl. Genet. 119: 507–517. 10.1007/s00122-009-1059-5 19449174 PMC2715469

[bib60] Arnon D. I, 1949 Copper enzymes in isolated choloroplasts. Polyphenol oxidase in Beta vulgaris. Plant Physiology 24: 1–15. 16654194 10.1104/pp.24.1.1 PMC437905

[bib61] Arjenaki F. G. Jabbaril R. Morshedi A., 2012 Evaluation of drought stress on relative water content, chlorophyll content and mineral elements of wheat (Triticum aestivum L.) varieties. International Journal of Agriculture and Crop Sciences 4: 726–729.

[bib4] Avni R. Nave M. Barad O. Baruch K. Twardziok S. O., 2017 Wild emmer genome architecture and diversity elucidate wheat evolution and domestication. Science 357: 93–97. 10.1126/science.aan0032 28684525

[bib62] Bars H. D. Weatherly P. E., 1962 A re-examination of the relative turgidity technique for estimating water deficits in leaves. Aust. J. Bios. Sci. 15: 415–418.

[bib63] Bates L. S. Waldren R. P. Tear I. D., 1973 Rapid determination of free proline for water stress studies. Plant and Soil 39: 205–207. 10.1007/BF00018060

[bib5] Beales J. Turner A. Griffiths S. Snape J. W. Laurie D. A., 2007 A Pseudo-Response Regulator is misexpressed in the photoperiod insensitive *Ppd-D1a* mutant of wheat (*Triticum aestivum* L.). Theor. Appl. Genet. 115: 721–733. 10.1007/s00122-007-0603-4 17634915

[bib64] Beauchamp C. Fridovich I., 1971 Superoxide dismutase improved assays and an assay applicable to acrylamide gels. Analytical Biochemistry 44: 276–287. 10.1016/0003-2697(71)90370-8 4943714

[bib6] Bevan M. W. Uauy C. Wulff B. B. Zhou J. Krasileva K., 2017 Genomic innovation for crop improvement. Nature 543: 346–354. 10.1038/nature22011 28300107

[bib7] Börner A. Ogbonnaya F. C. Röder M. S. Rasheed A. Periyannan S., 2015 *Aegilops tauschii* introgressions in wheat, Alien Introgression in Wheat, edited by Molnár-Láng M. Ceoloni C. Doležel J. Springer International, Switzerland 10.1007/978-3-319-23494-6_10

[bib8] Breseghello F. Sorrells M. E., 2006 Association mapping of kernel size and milling quality in wheat (Triticum aestivum L.) cultivars. Genetics 172: 1165–1177. 10.1534/genetics.105.044586 16079235 PMC1456215

[bib65] Cadzow M. Boocock J. Nguyen H. T. Wilcox P. Merriman T. P. Black M. A., 2014 A bioinformatics workflow for detecting signatures of selection in genomic data. Frontiers in Genetics 5: 293. 25206364 10.3389/fgene.2014.00293 PMC4144660

[bib9] Chen G. B. Lee S. H. Zhu Z. X. Benyamin B. Robinson M. R., 2016 EigenGWAS: finding loci under selection through genome-wide association studies of eigenvectors in structured populations. Heredity 117: 51–61. 10.1038/hdy.2016.25 27142779 PMC4901357

[bib66] Chen H. Patterson N. Reich D., 2010 Population differentiation as a test for selective sweeps. Genome Res 20: 393–402. 10.1101/gr.100545.109 20086244 PMC2840981

[bib10] Chen H. Iqbal M. Perez-Lara E. Yang R.-C. Pozniak C., 2015 Earliness per se quantitative trait loci and their interaction with Vrn-B1 locus in a spring wheat population. Mol. Breed. 35: 182 10.1007/s11032-015-0373-7

[bib11] Clavel D. Drame N. K. Roy-Macauley H. Braconnier S. Laffray D., 2005 Analysis of early responses to drought associated with field drought adaptation in four Sahelian groundnut (Arachis hypogaea L.) cultivars. Environ. Exp. Bot. 54: 219–230. 10.1016/j.envexpbot.2004.07.008

[bib12] Dreisigacker S. Tiwari R. Sheoran S., 2013 *Laboratory Manual: ICAR-CIMMYT Molecular Breeding Course in Wheat*. ICAR; BMZ; CIMMYT, Haryana, India.

[bib67] DuBois M. Gilles K. A. Hamilton J. K. Rebers P. A. Smith F., 1956 Colorimetric method for determination of sugars and related substances. Analytical Chemistry 28: 350–356.

[bib68] Evanno G. Regnaut S. Goudet J., 2005 Detecting the number of clusters of individuals using the software STRUCTURE: a simulation study. Molecular ecology 14: 2611–2620. 15969739 10.1111/j.1365-294X.2005.02553.x

[bib69] Giannopolitis C. N. Ries S. K., 1977 Superoxide dismutases. I. Occurrence in higher plants. Plant Physiology 59: 309–314. 10.1104/pp.59.2.309 16659839 PMC542387

[bib13] Hamblin M. T. Jannink J.-L., 2011 Factors affecting the power of haplotype markers in association studies. Plant Genome 4: 145–153. 10.3835/plantgenome2011.03.0008

[bib14] Hao C. Wang Y. Chao S. Li T. Liu H., 2017 The iSelect 9 K SNP analysis revealed polyploidization induced revolutionary changes and intense human selection causing strong haplotype blocks in wheat. Sci. Rep. 7: 41247 10.1038/srep41247 28134278 PMC5278348

[bib70] Hiscox J. D. Israelstam G. F., 1979 A method for the extraction of chlorophyll from leaf tissue without maceration. Canadian J Bot 57: 1332–1334.

[bib15] Hou J. Jiang Q. Hao C. Wang Y. Zhang H., 2014 Global selection on sucrose synthase haplotypes during a century of wheat breeding. Plant Physiol. 164: 1918–1929. 10.1104/pp.113.232454 24402050 PMC3982753

[bib71] Islam M. T. Uddin M. T. Sattar M. N., 2012 A comparative economic study on BARI gom-24 and BARI gom-23 production in a selected area of Dinajpur District. Progressive agriculture 23: 123–132.

[bib72] IWGSC I., 2018 Shifting the limits in wheat research and breeding using a fully annotated reference genome. Science 361: eaar7191. 30115783 10.1126/science.aar7191

[bib16] Jafarzadeh J. Bonnett D. Jannink J. L. Akdemir D. Dreisigacker S., 2016 Breeding value of primary synthetic wheat genotypes for grain yield. PLoS One 11: e0162860 10.1371/journal.pone.0162860 27656893 PMC5033409

[bib17] Jiang Y. Jiang Q. Hao C. Hou J. Wang L., 2015 A yield-associated gene TaCWI, in wheat: its function, selection and evolution in global breeding revealed by haplotype analysis. Theor. Appl. Genet. 128: 131–143. 10.1007/s00122-014-2417-5 25367379

[bib18] Jordan K. W. Wang S. Lun Y. Gardiner L. J. MacLachlan R., 2015 A haplotype map of allohexaploid wheat reveals distinct patterns of selection on homoeologous genomes. Genome Biol. 16: 48 10.1186/s13059-015-0606-4 25886949 PMC4389885

[bib19] Li X. P. Zhao X. Q. He X. Zhao G. Y. Li B., 2011 Haplotype analysis of the genes encoding glutamine synthetase plastic isoforms and their association with nitrogen-use- and yield-related traits in bread wheat. New Phytol. 189: 449–458. 10.1111/j.1469-8137.2010.03490.x 21039562

[bib20] Liu K. Muse S. V., 2005 PowerMarker: an integrated analysis environment for genetic marker analysis. Bioinformatics 21: 2128–2129. 10.1093/bioinformatics/bti282 15705655

[bib21] Lopes M. S. Reynolds M. P., 2011 Drought adaptive traits and wide adaptation in elite lines derived from resynthesized hexaploid wheat. Crop Sci. 51: 1617–1626. 10.2135/cropsci2010.07.0445

[bib22] Lorenz A. J. Hamblin M. T. Jannink J. L., 2010 Performance of single nucleotide polymorphisms *vs.* haplotypes for genome-wide association analysis in barley. PLoS One 5: e14079 10.1371/journal.pone.0014079 21124933 PMC2989918

[bib23] Luo M. C. Gu Y. Q. Puiu D. Wang H. Twardziok S. O., 2017 Genome sequence of the progenitor of the wheat D genome Aegilops tauschii. Nature 551: 498–502. 10.1038/nature24486 29143815 PMC7416625

[bib24] Luo M. C. Gu Y. Q. You F. M. Deal K. R. Ma Y. Q., 2013 A 4-gigabase physical map unlocks the structure and evolution of the complex genome of Aegilops tauschii, the wheat D-genome progenitor. Proc. Natl. Acad. Sci. USA 110: 7940–7945. 10.1073/pnas.1219082110 23610408 PMC3651469

[bib25] Ma D. Y. Yan J. He Z. H. Wu L. Xia X. C., 2012 Characterization of a cell wall invertase gene TaCwi-A1 on common wheat chromosome 2A and development of functional markers. Mol. Breed. 29: 43–52. 10.1007/s11032-010-9524-z

[bib26] Makumburage G. B. Stapleton A., 2011 Phenotype uniformity in combined-stress environments has a different genetic architecture than in single-stress treatments. Front. Plant Sci. 2: 12 10.3389/fpls.2011.00012 22645526 PMC3355809

[bib27] Merah O., 2001 Potential importance of water status traits for durum wheat improvement under Mediterranean conditions. J. Agric. Sci. 137: 139–145. 10.1017/S0021859601001253

[bib28] Morrell P. L. Buckler E. S. Ross-Ibarra J., 2011 Crop genomics: advances and applications. Nat. Rev. Genet. 13: 85–96. 10.1038/nrg3097 22207165

[bib29] Mujeeb-Kazi A. Rosas V. Roldan S., 1996 Conservation of the genetic variation of Triticum tauschii (Coss.) Schmalh. (Aegilops squarrosa auct. non L.) in synthetic hexaploid wheats (T. turgidum L. s.lat. x T. tauschii; 2n=6x=42, AABBDD) and its potential utilization for wheat improvement. Genet. Resour. Crop Evol. 43: 129–134. 10.1007/BF00126756

[bib30] Ogbonnaya F. C. Abdalla O. Mujeeb-Kazi A. Alvina G. K. Xu S. S., 2013 Synthetic hexaploids: harnessing species of the primary gene pool for wheat improvement. Plant Breed. Rev. 37: 35–122. 10.1002/9781118497869.ch2

[bib31] Ogbonnaya F. C. Rasheed A. Okechukwu E. C. Jighly A. Makdis F., 2017 Genome-wide association study for agronomic and physiological traits in spring wheat evaluated in a range of heat prone environments. Theor. Appl. Genet. 130: 1819–1835. 10.1007/s00122-017-2927-z 28577083

[bib32] Olsen K. M. Wendel J. F., 2013 A bountiful harvest: genomic insights into crop domestication phenotypes. Annu. Rev. Plant Biol. 64: 47–70. 10.1146/annurev-arplant-050312-120048 23451788

[bib73] Price A. L. Patterson N. J. Plenge R. M. Weinblatt M. E. Shadick N. A. Reich D., 2006 Principal components analysis corrects for stratification in genome-wide association studies. Nat. Genet. 38: 904–909. 16862161 10.1038/ng1847

[bib33] Purcell S. Neale B. Todd-Brown K. Thomas L. Ferreira M. A. R., 2007 PLINK: a tool set for whole-genome association and population-based linkage analyses. Am. J. Hum. Genet. 81: 559–575. 10.1086/519795 17701901 PMC1950838

[bib34] Qian L. Hickey L. T. Stahl A. Werner C. R. Hayes B., 2017 Exploring and harnessing haplotype diversity to improve yield stability in crops. Front. Plant Sci. 8: 1534 10.3389/fpls.2017.01534 28928764 PMC5591830

[bib35] Quraishi U. M. Pont C. Ain Q. U. Flores R. Burlot L., 2017 Combined genomic and genetic data integration of major agronomical traits in bread wheat (*Triticum aestivum* L.). Front. Plant Sci. 8: 1843 10.3389/fpls.2017.01843 29184557 PMC5694560

[bib36] Rasheed A. Hao Y. Xia X. C. Khan A. Xu Y., 2017 Crop breeding chips and genotyping platforms: progress, challenges and perspectives. Mol. Plant 10: 1047–1064. 10.1016/j.molp.2017.06.008 28669791

[bib37] Rasheed A. Mujeeb-Kazi A. Ogbonnaya F. C. He Z. H. Rajaram S., 2018 Wheat genetic resources in the post-genomics era: promise and challenges. Ann. Bot. (Lond.) 121: 603–616. 10.1093/aob/mcx148 PMC5852999 29240874

[bib38] Ray D. K. Mueller N. D. West P. C. Foley J. A., 2013 Yield trends are insufficient to double global crop production by 2050. PLoS One 8: e66428 10.1371/journal.pone.0066428 23840465 PMC3686737

[bib39] Reif J. C. Zhang P. Dreisigacker S. Warburton M. L. van Ginkel M., 2005 Wheat genetic diversity trends during domestication and breeding. Theor. Appl. Genet. 110: 859–864. 10.1007/s00122-004-1881-8 15690175

[bib40] Reynolds M. P. Mujeeb-Kazi A. Sawkins M., 2005 Prospects for utilising plant-adaptive mechanisms to improve wheat and other crops in drought- and salinity-prone environments. Ann. Appl. Biol. 146: 239–259. 10.1111/j.1744-7348.2005.040058.x

[bib41] Schmutz J. McClean P. E. Mamidi S. Wu G. A. Cannon S. B., 2014 A reference genome for common bean and genome-wide analysis of dual domestications. Nat. Genet. 46: 707–713. 10.1038/ng.3008 24908249 PMC7048698

[bib42] Spindel J. E. Begum H. Akdemir D. Collard B. Redona E., 2016 Genome-wide prediction models that incorporate de novo GWAS are a powerful new tool for tropical rice improvement. Heredity 116: 395–408. 10.1038/hdy.2015.113 26860200 PMC4806696

[bib43] Stephens J. C. Schneider J. A. Tanguay D. A. Choi J. Acharya T., 2001 Haplotype variation and linkage disequilibrium in 313 human genes. Science 293: 489–493. 10.1126/science.1059431 11452081

[bib44] Tang Y. Wu X. Li C. Yang W. Huang M., 2017 Yield, growth, canopy traits and photosynthesis in high-yielding, synthetic hexaploid-derived wheats cultivars compared with non-synthetic wheats. Crop Pasture Sci. 68: 115–125. 10.1071/CP16072

[bib45] Tilman D. Balzer C. Hill J. Befort B. L., 2011 Global food demand and the sustainable intensification of agriculture. Proc. Natl. Acad. Sci. USA 108: 20260–20264. 10.1073/pnas.1116437108 22106295 PMC3250154

[bib46] Voss-Fels K. P. Robinson H. Mudge S. R. Richard C. Newman S., 2017 VERNALIZATION1 modulates root system architecture in wheat and barley. Mol. Plant. 11: 226–229. 10.1016/j.molp.2017.10.005 29056533

[bib47] Wang S. C. Wong D. B. Forrest K. Allen A. Chao S. M., 2014 Characterization of polyploid wheat genomic diversity using a high-density 90 000 single nucleotide polymorphism array. Plant Biotechnol. J. 12: 787–796. 10.1111/pbi.12183 24646323 PMC4265271

[bib48] Wang S. W. Carver B. Yan L. L., 2009 Genetic loci in the photoperiod pathway interactively modulate reproductive development of winter wheat. Theor. Appl. Genet. 118: 1339–1349. 10.1007/s00122-009-0984-7 19234853

[bib49] Weir B. S. Cockerham C. C., 1984 Estimating F-statistics for the analysis of population structure. Evolution 38: 1358–1370. 28563791 10.1111/j.1558-5646.1984.tb05657.x

[bib50] Wilhelm E. P. Turner A. S. Laurie D. A., 2009 Photoperiod insensitive Ppd-A1a mutations in tetraploid wheat (Triticum durum Desf.). Theor. Appl. Genet. 118: 285–294. 10.1007/s00122-008-0898-9 18839130

[bib51] Wingen L. U. West C. Leverington-Waite M. Collier S. Orford S., 2017 Wheat landraces genome diversity. Genetics 205: 1657–1676. 10.1534/genetics.116.194688 28213475 PMC5378120

[bib52] Wu Z. Wang B. Chen X. Wu J. King G. J., 2016 Evaluation of linkage disequilibrium pattern and association study on seed oil content in *Brassica napus* using ddRAD sequencing. PLoS One 11: e0146383 10.1371/journal.pone.0146383 26730738 PMC4701484

[bib74] Xie W. Wang G. Yuan M. Yao W. Lyu K. Zhao H. Yang M. Li P. Zhang X. Yuan J. Wang Q., 2015 Breeding signatures of rice improvement revealed by a genomic variation map from a large germplasm collection. Proceedings of the National Academy of Sciences 112: 5411–5419. 10.1073/pnas.1515919112 PMC4593105 26358652

[bib53] Xu Y. Li P. Zou C. Lu Y. Xie C., 2017 Enhancing genetic gain in the era of molecular breeding. J. Exp. Bot. 68: 2641–2666. 10.1093/jxb/erx135 28830098

[bib75] Yu J Buckler E. S., 2006 Genetic association mapping and genome organization of maize. Current opinion in biotechnology 17: 155–160. 16504497 10.1016/j.copbio.2006.02.003

[bib76] Zadoks J. C. Chang T. T. Konzak C. F., 1974 A decimal code for the growth stages of cereals. Weed Research 14: 415–421.

[bib54] Zhang B. Liu X. Xu W. Chang J. Z. Li A., 2015 Novel function of a putative *MOC1* ortholog associated with spikelet number per spike in common wheat. Sci. Rep. 5: 12211 10.1038/srep12211 26197925 PMC4510493

[bib55] Zhang L. Zhao Y. L. Gao L. F. Zhao G. Y. Zhou R. H., 2012 TaCKX6–D1, the ortholog of rice OsCKX2, is associated with grain weight in hexaploid wheat. New Phytol. 195: 574–584. 10.1111/j.1469-8137.2012.04194.x 22670578

[bib56] Zhang Y. J. Liu J. D. Xia X. C. He Z. H., 2014 TaGS-D1, an ortholog of rice OsGS3, is associated with grain weight and grain length in common wheat. Mol. Breed. 34: 1097–1107. 10.1007/s11032-014-0102-7

[bib57] Zhao G. Zou C. Li K. Wang K. Li T., 2017 The *Aegilops tauschii* genome reveals multiple impacts of transposons. Nat. Plants 3: 946–955. 10.1038/s41477-017-0067-8 29158546

[bib58] Zheng J. Liu H. Wang Y. Wang L. Chang X., 2014 TEF-7A, a transcript elongation factor gene, influences yield-related traits in bread wheat (*Triticum aestivum* L.). J. Exp. Bot. 65: 5351–5365. 10.1093/jxb/eru306 25056774 PMC4157721

[bib59] Zikhali M. Wingen L. U. Griffiths S., 2016 Delimitation of the Earliness per se D1 (Eps-D1) flowering gene to a subtelomeric chromosomal deletion in bread wheat (Triticum aestivum). J. Exp. Bot. 67: 287–299. 10.1093/jxb/erv458 26476691 PMC4682435

